# Targeting vulnerabilities in IDH mutant tumours: The model matters

**DOI:** 10.1016/j.neo.2026.101338

**Published:** 2026-07-27

**Authors:** Alwine B. Kruisselbrink, Tessa A.H. Wilpshaar, Ieva Palubeckaitė, Romy C. Dijkland, Tatiana Belova, Sara Cardoso, Pauline M. Wijers-Koster, Inge H. Briaire-de Bruijn, René J.M. van Zeijl, Hans Dalebout, Marieke L. Kuijjer, Hailiang Mei, Bram Heijs, Karoly Szuhai, Judith V.M.G. Bovée, Sanne Venneker

**Affiliations:** a Department of Pathology, Leiden University Medical Center, Leiden, The Netherlands; b Center for Proteomics & Metabolomics, Leiden University Medical Center, Leiden, The Netherlands; c Centre for Molecular Medicine Norway (NCMM), Nordic EMBL Partnership, University of Oslo, Oslo, Norway; d iCAN Digital Precision Cancer Medicine Flagship, University of Helsinki and Helsinki University Hospital, Helsinki, Finland; e Department of Biochemistry and Developmental Biology, University of Helsinki, Helsinki, Finland; f Sequencing Analysis Support Core, Leiden University Medical Center, Leiden, The Netherlands; g Department of Cell and Chemical Biology, Leiden University Medical Center, Leiden, The Netherlands; h Department of Pathology, Netherlands Cancer Institute, Amsterdam, The Netherlands

**Keywords:** IDH1/IDH2 mutations, D-2-hydroxyglutarate, synthetic lethality, *in vitro* models, CRISPR-Cas9, chondrosarcoma

## Abstract

**Introduction:**

Synthetic lethal interactions with IDH1 and IDH2 (IDH) mutations were identified in non-endogenous IDH mutant (IDH^MUT^) AML and glioma models, but are absent in endogenous IDH^MUT^ chondrosarcoma cell lines. The translation into successful clinical applications has remained challenging, implying artificially created models do not fully recapitulate endogenous IDH^MUT^ tumour biology. The aim of this study was to elucidate if the model system is indeed an important factor to consider when studying therapeutic vulnerabilities in IDH^MUT^ tumours.

**Methods:**

Vector-based and CRISPR-Cas9 approaches were used to introduce or revert the IDH1 mutation in chondrosarcoma cell lines. These isogenic cell line pairs were used to examine the presence of known therapeutic vulnerabilities and their underlying biological mechanisms.

**Results:**

Vector-based IDH^MUT^ chondrosarcoma models showed the previously reported synthetic lethal interactions, but these treatment sensitivities were absent in the CRISPR-edited models. Interestingly, not all vector-based IDH^MUT^ cell lines displayed the same therapeutic vulnerabilities. Differences in treatment response were associated with multiple factors, including IDH^MUT^ protein expression and D-2-HG levels, in line with the fact that therapeutic vulnerabilities could be induced in the CRISPR-edited models by enhancing D-2-HG levels.

**Conclusion:**

Our findings demonstrate that synthetic lethal interactions observed in vector-based models are often a consequence of IDH^MUT^ protein overexpression and supra-physiological D-2-HG levels. These results highlight that relying on artificially created IDH^MUT^ models may lead to the identification of therapeutic vulnerabilities that are not present in IDH^MUT^ tumours, potentially explaining the poor translation of preclinical findings to clinical trials.

## Highlights

• The model system influences therapeutic vulnerabilities in IDH^MUT^ cell lines.
• Therapeutic vulnerabilities in non-endogenous IDH^MUT^ models may be model artifacts.
• IDH^MUT^ overexpression and high D-2-HG levels induce treatment sensitivities.
• Non-endogenous IDH^MUT^ models can generate false positive therapeutic leads.
• Use of endogenous IDH^MUT^ models is recommended for clinical translation.

## Introduction

Recurrent heterozygous point mutations in the isocitrate dehydrogenase 1 and -2 (IDH1 and IDH2) genes were identified as potential driver mutations in multiple tumour types, including chondrosarcoma, acute myeloid leukaemia (AML), glioma, and cholangiocarcinoma. The prevalence of IDH1 and IDH2 mutations ranges from ∼10-20% (AML and cholangiocarcinoma) to ∼50% in chondrosarcoma and >70% in glioma [[1], [2], [3]]. IDH1 and IDH2 (collectively referred to as IDH) mutations typically occur in the arginine residues at p.R132 and p.R140/p.R172, respectively, but certain variants are more common in specific tumour types ([4],[5]). The mutated IDH enzymes acquire a neomorphic function to metabolize α-ketoglutarate (α-KG) into the oncometabolite D-2-hydroxyglutarate (D-2-HG), although the produced quantities of D-2-HG differ between specific variants [[6], [7], [8]]. The most common variant in chondrosarcoma and cholangiocarcinoma (i.e., p.R132C) is classified as an efficient D-2-HG producer, whilst p.R140Q and p.R132H, the most common variants in AML and glioma respectively, produce lower quantities of the oncometabolite. D-2-HG is a competitive inhibitor of α-KG-dependent enzymes [9] and can thus cause a wide variety of biological changes, including alterations in the epigenetic and metabolic landscape, defects in DNA repair processes, and changes in growth signalling pathways [10].

Despite the broad downstream biological effects of IDH mutations and their presumed role in driving tumourigenesis, clinical trials with IDH mutant (IDH^MUT^) inhibitors showed limited clinical benefit in most patients, except those with low grade glioma or AML [[11], [12], [13], [14], [15], [16]]. However, novel acquired mutations in receptor tyrosine kinases (RTKs), amongst other mechanisms, can cause resistance in initially responding AML patients [17]. This aligns with the lack of response in a subset of chondrosarcoma patients with additional RTK pathway mutations [18]. Together, these findings suggest that some tumours lose dependency on their IDH mutation over time, which can be caused by additionally acquired mutations that are more dominant in driving tumourigenesis. Therefore, recent preclinical studies mainly focussed on directly targeting the underlying vulnerabilities of IDH^MUT^ tumours. Due to the limited availability of endogenous IDH^MUT^ AML and glioma cell lines, most studies relied on models in which the IDH mutation was introduced by lentiviral transduction, whereas chondrosarcoma studies used endogenous IDH wildtype (IDH^WT^) and IDH^MUT^ cell lines [19]. Synthetic lethal interactions with the IDH mutation, including radiotherapy, PARP inhibition, and Bcl-2 family member inhibition, were identified in the studies on AML, glioma, and cholangiocarcinoma [[19], [20], [21], [22], [23], [24], [25], [26]]. On the contrary, endogenous IDH^WT^ and IDH^MUT^ chondrosarcoma cell lines did not show a significant difference in treatment response, and short- or long-term exposure to an IDH1^MUT^ protein inhibitor (e.g., AGI-5198) could not rescue the treatment effects ([19],[[27], [28], [29], [30]]).

While the identification of synthetic lethal interactions with IDH mutations initially offered promising therapeutic avenues, their translation into effective clinical applications has remained challenging. This suggests that artificially created IDH^MUT^ models might not fully recapitulate the downstream biological effects of endogenous IDH mutations. The aim of the present study was to elucidate if the utilized model system (endogenous vs. genetically modified) is indeed an underlying denominator in defining therapeutic vulnerabilities in IDH^MUT^ cell lines, and could therefore lead to false positive therapeutic discoveries with limited clinical relevance.

## Materials and Methods

### Cell culture

The central conventional chondrosarcoma cell line OUMS27 (IDH^WT^) ([3],[31]) was cultured in RPMI medium (Gibco, Invitrogen, Scotland, UK) with 20% heat-inactivated fetal bovine serum (FBS) (F7524, Sigma-Aldrich, Saint Louis, MO, USA). All other chondrosarcoma cell lines, including their genetically modified derivatives, were cultured as previously described [32]. All cells were cultured in a humidified incubator with 5% CO_2_ at 37°C. A PCR-based mycoplasma test and Short Tandem Repeat (STR) profiling (GenePrint 10 System, Promega, Madison, WI, USA) were performed regularly.

### Compounds

RO-3306 (CDK1 inhibitor, Selleck Chemicals, Houston, TX, USA), ABT-737 (Bcl-2/Bcl-xL/Bcl-w inhibitor, Selleck Chemicals), AGI-5198 (IDH1^MUT^ protein inhibitor, Cayman Chemical, Ann Arbor, MI, USA), and talazoparib (PARP inhibitor, Selleck Chemicals) were dissolved in DMSO. Cell permeable D-2-HG ((2R)-Octyl-α-hydroxyglutarate, 16336, Cayman Chemical) was freshly dissolved in PBS. All compounds were dissolved according to the manufacturer’s protocol.

### Establishment and culturing of vector-based CH2879 IDH1^R132C^ cell lines

The empty vector (EV) and the IDH1^R132C^ vector were cloned as described in the Supplementary Material and Methods. Vectors contain the endogenous IDH1 promoter to drive expression of either EGFP alone or bicistronic expression of EGFP and IDH1^MUT^, with the sequences separated by P2A/T2A sites to enable expression of two individual proteins from a single RNA transcript (Supplementary Fig. S1A). The chondrosarcoma cell line CH2879 (IDH^WT^) was seeded, allowed to attach overnight, and transduced with 8 µg/mL polybrene and lentivirus of interest. Lentiviral particles were produced as described in the Supplementary Material and Methods. After overnight incubation, medium was replaced, and cells were cultured for several passages until fluorescence-activated cell sorting (FACS) on EGFP expression using the BD FACS Aria II 3L (BD Biosciences, San Jose, CA, USA). CH2879 cells transduced with the EV were sorted in an EGFP-positive fraction (CH2879 EV polyclonal cell population), whilst cells transduced with the IDH1^R132C^ vector were sorted in fractions weakly and strongly positive for EGFP expression (CH2879 low and high IDH1^R132C^ copy number polyclonal cell populations, respectively) (Supplementary Fig. S2). For clonal expansion, CH2879 IDH1^R132C^ polyclonal cell populations were single cell sorted into 96-well plates. In total, four low IDH1^R132C^ copy number (CN) single cell clones and six high IDH1^R132C^ CN single cell clones were established. Treatment response was evaluated at 7 to 50 passages after introduction of the IDH mutation, providing ample time for epigenetic remodeling [33].

### Establishment and culturing of CRISPR-Cas9 gene edited CH2879 IDH1^R132C^ and JJ012 IDH1^WT^ cell lines

The chondrosarcoma cell lines CH2879 (IDH^WT^) and JJ012 (IDH1^R132G^) were reverse transfected with the Edit-R hCMV-PuroR-Cas9 plasmid (Horizon Discovery, Cambridge, UK) and Lipofectamine Stem Transfection Reagent (Invitrogen). After overnight incubation, transfected cells were selected with 4 µg/mL puromycin. To enhance homology directed repair (HDR) efficiency, Cas9 expressing cells were treated with 7.5 µM RO-3306 for 16 hours to induce a G2/M-phase arrest. Arrested cells were harvested and reverse transfected with tracrRNA (Horizon Discovery), an IDH1 crRNA (Supplementary Table S1, Horizon Discovery), an IDH1 homology arm (Supplementary Table S1, Integrated DNA Technologies, Coralville, IA, USA), and DharmaFECT 3 (CH2879 cells) or DharmaFECT 1 (JJ012 cells) (Horizon Discovery). IDH1 crRNAs were designed around the IDH1 p.R132 target site and homology arms (150 bp) contained point mutations to introduce an IDH1 p.R132R or p.R132C variant, silent PAM mutations to inhibit Cas9 cleavage, and a novel PsiI restriction site to verify HDR (Supplementary Fig. S1B and Supplementary Table S1). Limiting dilutions were performed to establish single cell clones. In total, two CH2879 IDH1^R132C^ and four JJ012 IDH1^WT^ single cell clones were established. As a control, four mock single cell clones were established for each parental cell line. These mock controls underwent the same experimental procedure, but without addition of tracrRNA, crRNA, and a homology arm to the transfection mixes. Treatment response was evaluated at 13 to 38 passages after introduction or reversion of the IDH mutation, providing ample time for epigenetic remodeling [33].

### Detection of IDH1^R132C^ vector copy numbers

DNA was isolated with the Wizard Genomic DNA Purification Kit (Promega) according to the manufacturer’s protocol and eluted in 10 mM Tris-HCl pH 8.0 with 0.1 mM EDTA. Multiplex Ligation-dependent Probe Amplification (MLPA) assays were performed using the P088 oligodendroglioma 1p-19q probe mix and EK1-FAM reagent kit (MRC Holland, Amsterdam, the Netherlands) according to the manufacturer’s protocol (version 7). Data analysis was performed with Coffalyser.Net (v.210226.1433, MRC Holland). The binning sample was used to link probe signals to specific genes and data were normalized to reference probes (n=25) and probe signals of HT1080 cells (IDH1^R132C^) to obtain probe ratio values. L835 cells (IDH1^R132C^) and CH2879 cells (IDH^WT^) were included in all assays as positive and negative controls, respectively. For each cell line, three independent samples were measured in triplicate.

### Measurement of D-2-HG levels with liquid chromatography-tandem mass spectrometry

To each cell pellet (0.5 to 1 × 10^6^ cells), 5 nmol Disodium (RS)-2-Hydroxy-1,5-pentanedioate-2,3,3-d_3_,OD (D-7496, C/D/N Isotopes, Quebec, Canada) was added as internal standard (IS). Metabolites were extracted with ice-cold 89% methanol (LC-MS grade, diluted in Milli-Q water), followed by ultrasonication, protein precipitation at -20°C, and collection of supernatants. Steps were repeated to ensure proper metabolite extraction.

Standards of L-2-HG/D-2-HG (61313 and 61382, Sigma-Aldrich) were prepared in 80% methanol + 0.1 mM lactate containing 0 to 15 nmol of the individual enantiomers and 5 nmol IS. Derivatization of samples and standards was performed as previously described [34], but Diacetyl-L-Tartaric Anhydride (DATAN, 358924, Sigma-Aldrich) treatment was extended (2 h at 75°C) and final sample residues were dissolved in 20% acetic acid (LC-MS grade, diluted in Milli-Q water). Liquid chromatography-tandem mass spectrometry (LC-MS/MS) was performed as previously described [34]. D-2-HG peak areas of standards and samples were divided by IS peak areas and an IS-corrected standard curve was used for calculations. Values were normalized to cell number to obtain nmol D-2-HG per 1 × 10^6^ cells. HT1080 and L835 cells were included as reference samples for D-2-HG production by endogenous IDH1 p.R132C mutations. CH2879 cells (IDH^WT^) and JJ012 cells (IDH1^R132G^) were included in all measurements as positive and negative controls, respectively. For each cell line, three independent samples were measured.

### Fluorometric D-2-HG assay

To prepare cellular extracts, 1 × 10^6^ cells were washed twice with ice-cold PBS, lysed in 200 µL 1% SDS/0.5 M EDTA/1 M Tris pH 7.4 in Milli-Q water, and homogenized with QIAshredder columns (Qiagen, Venlo, the Netherlands) according to the manufacturer’s protocol. Cellular extracts (75 µL) were transferred to a 96-well plate and deproteinized by 4 M perchloric acid (PCA, Sigma-Aldrich) according to the standard protocol of the D-2-HG Assay Kit (MAK320, Sigma-Aldrich), except pH neutralization by 2 M potassium hydroxide (KOH, Sigma-Aldrich) was performed with a volume equalizing 60% of the PCA-treated supernatant. D-2-HG was detected in 25 µL of KOH-treated supernatants and standard curve samples according to the manufacturer’s protocol. The standard curve was used to calculate the amount of pmol D-2-HG in the samples. These values correspond to approximately 6 × 10^5^ cells, as only a fraction of the original cellular extract was analysed. Three independent samples were measured per cell line.

### Western Blotting

Preparation of protein lysates, western blotting, imaging, and quantification of protein expression levels were performed as previously described [29]. Western blots were examined for the proteins listed in Supplementary Table S2.

### Population doubling times

For the vector-based CH2879 IDH1^R132C^ cell lines, population doubling times were extracted from the GR Calculator dose-response curve data (see below for methods). For the CRISPR-edited cell lines, cells were seeded in 96-well plates in different densities (5,000 to 10,000 cells/well) and nuclei count assays were performed one and four days after seeding as previously described [29]. Population doubling times were calculated with the following formula: duration x log10(2) / log10(final cell count) – log10(initial cell count). Conditions in which no linear growth was observed were excluded. Experiments were performed two times in triplicate.

### Dose-response curves

Cells were seeded in 96-well plates (3,000 to 10,000 cells/well), allowed to attach overnight, and treated for 72 h with the compounds mentioned above in concentrations ranging from 1 nM to 31.6 µM. DMSO or PBS treatment were included as vehicle controls. Nuclei count assays to determine drug response were performed and analysed as previously described [29]. Data were normalized to the vehicle control and “time 0 measurements” to correct for differences in growth rate kinetics between cell lines (GR Calculator, http://www.grcalculator.org). Experiments were performed at least three times in triplicate.

### Radiation response by colony formation assays

Cells were seeded in 10 cm dishes in densities ranging from 400 to 14,400 cells per dish. After overnight attachment, cells were irradiated at 0, 2, 4 or 6 Gy of y-radiation using a ^137^C source (YXLON, Comet Technologies, Shelton, CT, USA). Colonies were allowed to form for two to three weeks. Dishes were stained and analysed as previously described [29]. Data were normalized to the plating efficiency of the corresponding untreated control to obtain surviving fractions. Experiments were performed at least two times in duplicate.

### Statistical Analysis

ROUT’s tests (Q = 1%) were performed to detect outlier values in the dose-response curve datasets. Kruskal–Wallis tests followed by Dunn’s post-hoc tests were performed to detect statistically significant differences between experimental groups. Spearman correlations were performed to determine statistically significant associations between two variables. All statistical tests were performed at α = 0.05 using GraphPad Prism (Version 10.6.1, GraphPad Software, San Diego, CA, USA).

### Supplementary Materials and Methods

For descriptions of cell cycle analysis, karyotyping, Sanger sequencing, whole exome sequencing, and RNA sequencing, see the Supplementary Material and Methods.

## Results

### Vector-based CH2879 IDH1^MUT^ cell lines exhibit heterogeneous IDH1^R132C^ expression and D-2-HG production that can exceed endogenous levels

**Fig. 1:**
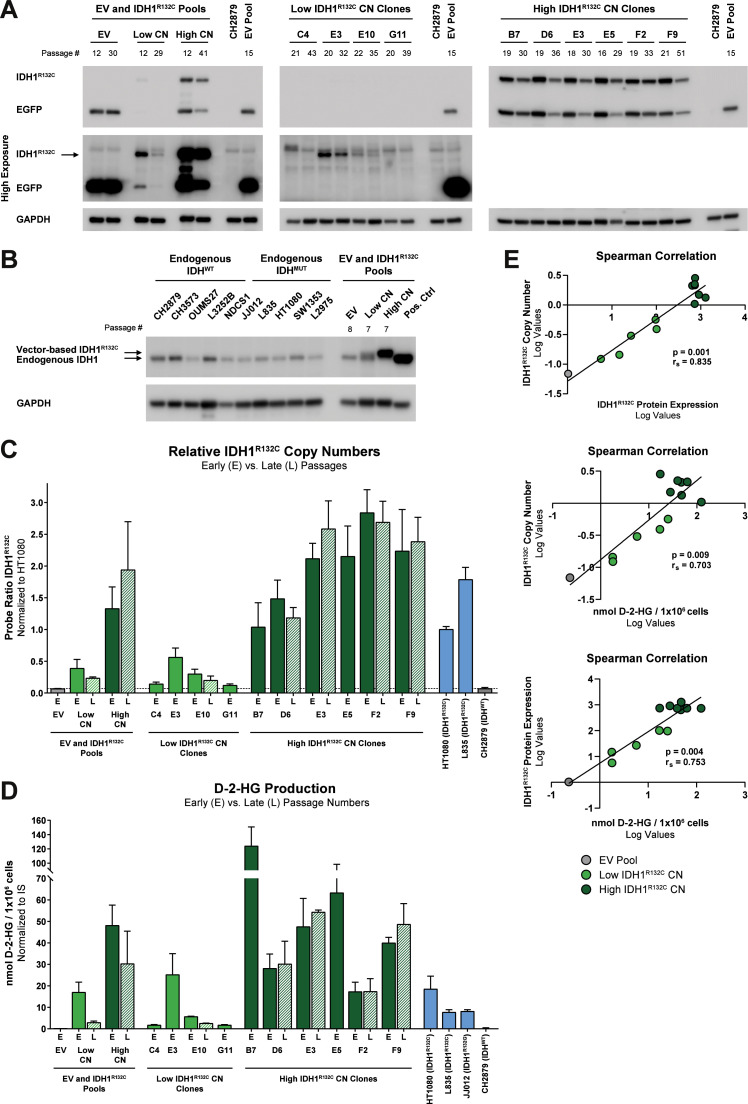
Vector-based CH2879 IDH1^MUT^ cell lines exhibit heterogeneous IDH1^R132C^ expression and D-2-HG production that can exceed endogenous levels. (A) Western blots for IDH1 p.R132C and EGFP expression, both detected with a T2A/P2A antibody. IDH1^R132C^ and EGFP protein expression were not visible by eye for all low IDH1^R132C^ CN clones, but a signal for both proteins was detected in all cell lines by quantification (Supplementary Fig. S3A-B). Passage numbers indicate the number of passages after lentiviral transduction. CH2879 and EV p15 cells were included as negative controls. GAPDH expression was used as a loading control. **(B)** Western blot for total IDH1 expression in endogenous and artificially created IDH^WT^ and IDH1^MUT^ chondrosarcoma cell lines. Arrows indicate endogenous IDH1 and vector-based IDH1^R132C^ expression. The latter is slightly larger due to inclusion of T2A/P2A sites in the protein. Passage numbers indicate the number of passages after lentiviral transduction. HepG2 cells were included as a positive control. GAPDH expression was used as a loading control. **(C)** MLPA assays were performed to obtain the number of integrated IDH1^R132C^ vector copies relative to HT1080 cells. Passage numbers are comparable to those depicted in panel A. L835 and CH2879 cells were included as positive and negative controls, respectively. Bars represent the mean ± standard deviation of three independent samples measured in triplicate. A Kruskal–Wallis tests followed by Dunn’s post-hoc tests did not detect statistically significant changes (p ≤ 0.05) between early (E) and late (L) passages of individual cell lines. **(D)** D-2-HG production per one million cells measured by LC-MS/MS. Passage numbers are comparable to those depicted in panel A. HT1080 and L835 cells were included as reference samples for D-2-HG production by endogenous IDH1 p.R132C mutations. CH2879 and JJ012 cells were included as negative and positive controls, respectively. Bars represent the mean ± standard deviation of three independent samples measured one time. A Kruskal–Wallis tests followed by Dunn’s post-hoc tests did not detect statistically significant changes (p ≤ 0.05) between early (E) and late (L) passages of individual cell lines. **(E)** Spearman correlations of IDH1^R132C^ copy number, IDH1^R132C^ protein expression, and D-2-HG production. Values were log transformed. All parameters showed a significant positive correlation (α = 0.05) with spearman’s rank correlation coefficients (r_s_) >0.7.

The IDH^WT^ chondrosarcoma cell line CH2879 was transduced with the empty vector (EV) or the IDH1^R132C^ vector (Supplementary Fig. S1A) and sorted on EGFP expression to establish the EV and relatively low and high IDH1^R132C^ copy number (CN) polyclonal cell populations (Supplementary Fig. S2), from which single cell clones were derived. Established IDH1^MUT^ cell lines showed on average an increase in population doubling time (∼30%) as compared to the parental (Table 1), implying an IDH mutation does not induce proliferation. The number of integrated IDH1^R132C^ vector copies, IDH1^R132C^ protein expression, and D-2-HG production varied substantially between established cell lines (Table 1, Fig. 1A-D, and Supplementary Fig. S3A-B), but were significantly positively associated with one another (Fig. 1E). Importantly, some artificially created IDH1^MUT^ models, predominantly high IDH1^R132C^ CN cell lines, showed higher total IDH1 expression (average increase of 4-fold) and D-2-HG levels (average increase of 3-fold) as compared to endogenous chondrosarcoma cell lines (Fig. 1B, 1D, Table 1, and Supplementary Fig. S3C). Established cell lines remained IDH1^MUT^ whilst culturing over time, without statistically significant changes in copy number, protein expression, or D-2-HG production (Fig. 1A, 1C-D, and Supplementary Fig. S3A). Hence, a unique panel of artificially created IDH1^MUT^ chondrosarcoma cell lines with distinct characteristics was established (see overview in Table 1), providing a platform to explore if therapeutic vulnerabilities differ between endogenous versus non-endogenous, vector-based IDH^MUT^ models.

**Table 1:** Characteristics of early passages of vector-based CH2879 EV and IDH1^R132C^ cell lines reveals a wide range of IDH1^MUT^ expression and therapeutic sensitivities.

		**Doubling time** Days	**IDH1^R132C^ CN** relative to HT1080	**IDH1^R132C^ protein expression** relative to EV	**D-2-HG** nmol	**ABT-737 GR_50_** µM	**Talazoparib GR_50_** µM	**SF_2_**
**Pools**	**EV**	1.5	0.07	1	0.23	4.65	2.94	0.45
	**Low IDH1^R132C^ CN**	1.6	0.39	101	17.1	1.28	1.40	0.22
	**High IDH1^R132C^ CN**	1.9	1.33	1279	48.2	0.54	0.66	0.20
**Clones** Low IDH1^R132C^ CN	**C4**	1.4	0.14	15	1.78	2.04	1.46	0.45
	**E3**	1.6	0.57	96	25.2	3.16	6.60	0.55
	**E10**	1.8	0.30	27	5.72	1.15	2.00	0.35
	**G11**	1.5	0.12	6	1.79	2.98	2.27	0.30
**Clones** High IDH1^R132C^ CN	**B7**	2.4	1.04	729	124	0.35	0.70	N/D*
	**D6**	4.1	1.49	909	28.2	0.20	0.47	0.23
	**E3**	1.7	2.12	641	47.6	2.38	2.05	0.21
	**E5**	2.0	2.15	737	63.4	0.94	1.38	0.12
	**F2**	2.1	2.84	745	17.3	0.30	1.41	0.10
	**F9**	1.9	2.24	730	40.0	0.76	2.72	0.21

GR_50_ = growth rate corrected equivalent of the IC_50_ value; SF_2_ = surviving fraction at 2 Gy; N/D = not determined; * = Clone B7 high IDH1^R132C^ CN did not form colonies.

### Therapeutic sensitivity in vector-based IDH1^MUT^ models associates with the level of IDH1^MUT^ overexpression

**Fig. 2:**
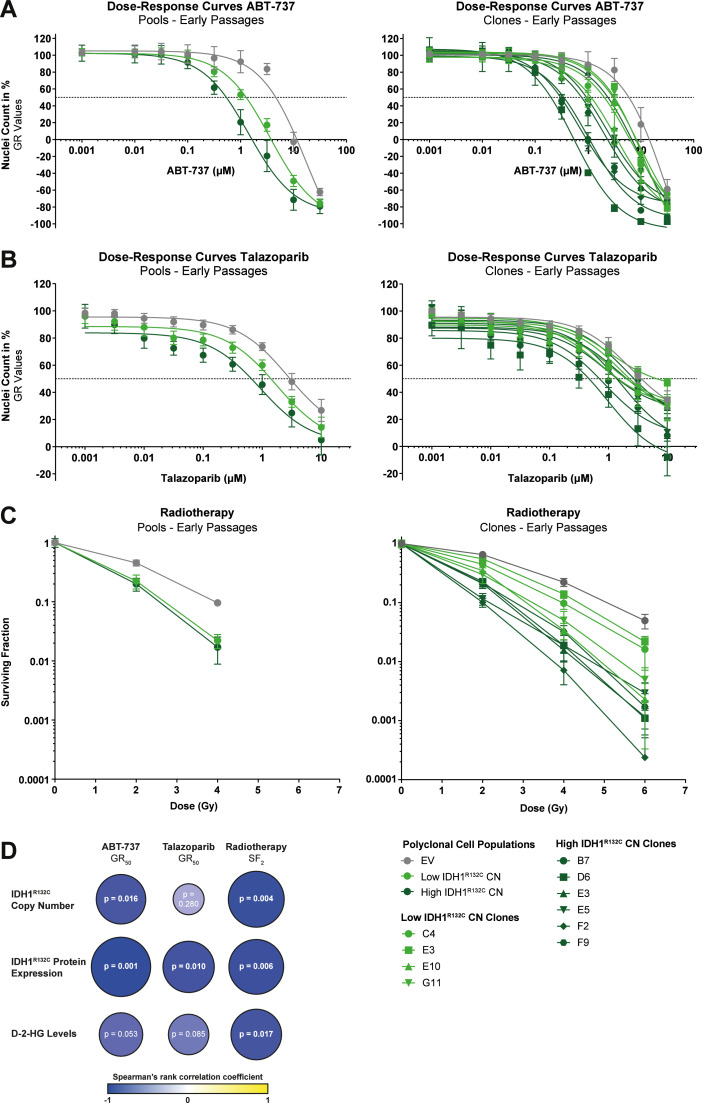
Therapeutic sensitivity in vector-based IDH1^MUT^ models associates with the level of IDH1^MUT^ overexpression. Dose-response curves of three days **(A)** ABT-737 (Bcl-2/Bcl-xL/Bcl-w inhibitor) and **(B)** talazoparib (PARP inhibitor) treatment. Data were corrected for growth rate differences between cell lines (GR values) and GR_50_ values are listed in Table 1. Data points represent the mean ± standard deviation of three independent experiments performed in triplicate. **(C)** Surviving fractions after treatment with single dosages of γ-radiation. One high IDH1^R132C^ CN single cell clone (B7) showed no outgrowth of colonies in the untreated control. SF_2_ values are listed in Table 1. Data points represent the mean ± standard deviation of two independent experiments performed in duplicate. **(D)** Spearman correlations of IDH1^R132C^ copy number, IDH1^R132C^ protein expression, and D-2-HG production versus treatment response (GR_50_ and SF2 values). Heatmap colours and circle size represent the spearman’s rank correlation coefficients, ranging from -1 (blue) to 1 (yellow) with larger circles representing a stronger positive or negative correlation. Obtained p-values are listed in the circles of the heatmap.

Most vector-based IDH1^MUT^ cell lines were more sensitive to ABT-737 (Bcl-2/Bcl-xL/Bcl-w inhibitor), talazoparib (PARP inhibitor), and radiotherapy than the EV pool (Fig. 2A-C, Table 1). Average reduction of growth rate corrected IC_50_ (GR_50_) or surviving fractions at 2 Gy (SF_2_) values was ∼70%, ∼30%, and ∼40% respectively, although these differences were not statistically significant. These synthetic lethal interactions were previously not observed in endogenous IDH^MUT^ chondrosarcoma cell lines ([19,[27], [28], [29], [30]]). Treatment with the IDH1^MUT^ protein inhibitor AGI-5198 only partially rescued treatment effects in the polyclonal IDH1^MUT^ cell populations (Supplementary Fig. S4A-C), which might be ascribed to residual D-2-HG production at a level (∼8 nmol/1 × 10^6^ cells) which was as high as D-2-HG levels in the endogenous IDH1^MUT^ JJ012 cell line (Supplementary Fig. S4D). Remarkably, the various IDH1^MUT^ clones showed a heterogenous therapy response (good versus partial responders), and some cell lines were intrinsically resistant towards treatment (Fig. 2A-C, Table 1) or acquired resistance whilst culturing over time (Supplementary Fig. S5 and S6). This indicated that other factors than the IDH mutation status influenced treatment response. IDH1^R132C^ copy numbers and protein expression, as well as D-2-HG levels, showed a significant negative association with treatment response (Fig. 2D), implying overexpression of the IDH mutation underlies the observed synthetic lethal interactions. However, some high IDH1^MUT^ CN clones (i.e., E3 and F9) were only partially sensitive to ABT-737 and talazoparib treatment respectively (∼50% and ∼10% reduction in GR_50_) (Table 1), indicating other factors also play a role in shaping treatment response. Indeed, extensive variation in the expression of both anti- and pro-apoptotic Bcl-2 family members between IDH1^MUT^ clones as well as within cell lines over time was observed (Supplementary Fig. S7A). PARP and ATM expression were reduced in some of the IDH1^MUT^ clones as compared to the EV pool (Supplementary Fig. S7B). Relative expression levels of Mcl-1, Bax, and all investigated pro-apoptotic proteins combined associated significantly with ABT-737 treatment response and ATM expression was the strongest predictor for radiotherapy sensitivity (Supplementary Fig. S7C). Transcriptome sequencing of a subset of cell lines showed distinct pathway activity patterns for individual IDH1^MUT^ cell lines when compared to the EV pool and revealed that biological differences between IDH1^MUT^ cell lines go beyond apoptotic and DNA repair pathways (Supplementary Fig. S8). Moreover, some IDH1^MUT^ cell lines rewired their biological activity over time, which could underly the observed acquired therapy resistance although no common resistance mechanisms were identified (Supplementary Fig. S9). Hence, the type of *in vitro* model (endogenous versus artificially created) as well as individual characteristics of vector-based IDH1^MUT^ cell lines determine if synthetic lethal interactions with the IDH mutation are present or not. Downstream biological changes induced by the IDH1 mutation can differ between and within vector-based IDH1^MUT^ cell lines, which could lead to the identification of therapeutic targets that are normally not present in IDH^MUT^ tumours and thus have limited clinical relevance.

### Chondrosarcoma cells do not necessarily depend on their endogenous IDH1 mutation for survival

**Fig. 3:**
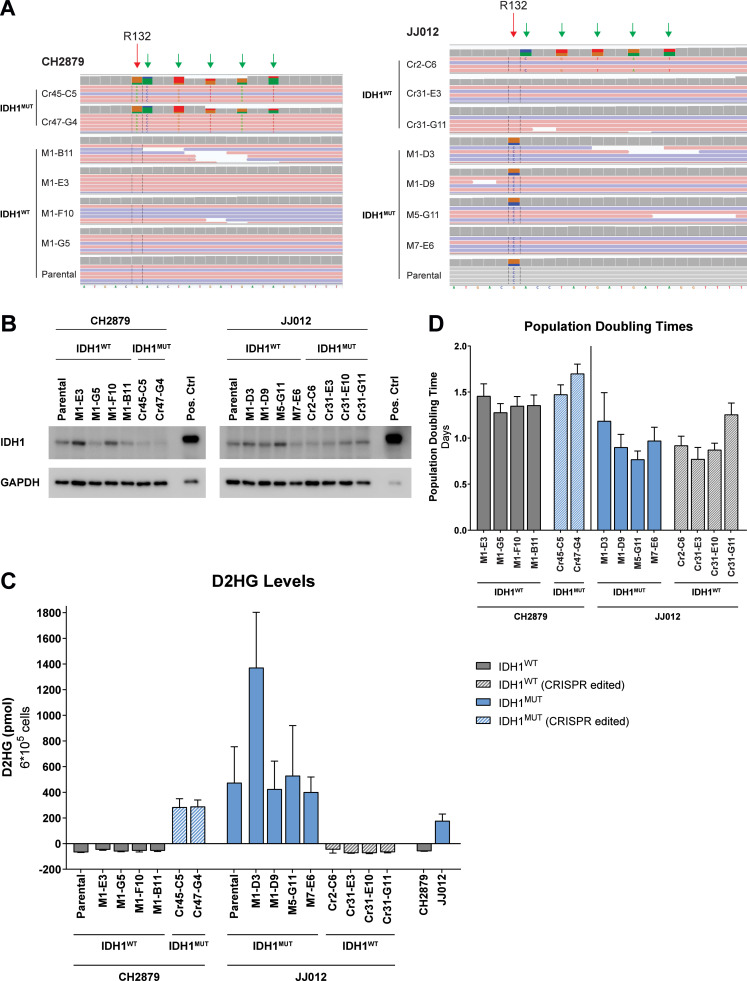
Chondrosarcoma cells do not necessarily depend on their endogenous IDH1 mutation for survival. (A) Whole exome sequencing reads showing the IDH1 mutational status around the R132 CRISPR-Cas9 target site in the established mock and CRISPR-edited single cell clones. The red arrow indicates the R132 target site, and the green arrows indicate the expected introduced silent mutations after homology directed repair. JJ012 Cr31-E3 and Cr31-G11 cells used their sister chromatid to repair their IDH1 p.R132G mutation, resulting in a lack of the expected silent mutations. WES of JJ012 Cr31-E10 failed and the IDH1 mutational status was verified by Sanger sequencing (Supplementary Fig. S10A). **(B)** Expression of total IDH1 in parental, mock, and CRISPR-edited IDH^WT^ and IDH1^MUT^ chondrosarcoma cell lines. HepG2 cells were included as a positive control. GAPDH expression was used as a loading control. **(C)** D-2-HG production per 6 × 10^5^ cells measured with the fluorometric D-2-HG assay. CH2879 and JJ012 parental cells were included as negative and positive controls, respectively. Bars represent the mean ± standard deviation of three independent samples measured one time. **(D)** Population doubling times in days for mock and CRISPR-edited IDH^WT^ and IDH1^MUT^ chondrosarcoma cell lines. Bars represent the mean ± standard deviation of two independent experiments performed in triplicate.

As IDH1^MUT^ overexpression was one of the factors underlying the variable treatment response in vector-based IDH1^MUT^ chondrosarcoma cell lines, a homology-directed repair CRISPR-Cas9 gene editing strategy was used to control IDH1^MUT^ copy numbers (Supplementary Fig. S1B). A similar CRISPR-Cas9 gene editing strategy was used to revert an endogenous IDH1 p.R132G mutation to a WT allele to examine if chondrosarcoma cells depend on their IDH1^MUT^ for survival. Whole exome sequencing (WES) and Sanger sequencing confirmed the successful introduction or reversion of IDH1^MUT^ in the established cell lines (Fig. 3A and S10A). CH2879 IDH1^MUT^ single cell clones retained at least one unedited WT allele and had an IDH1^MUT^ variant allele frequency (VAF) of 22-24% (Supplementary Table S3), in line with the hypotriploid karyotype of the CH2879 cell line with three to four copies of chromosome 2 [35]. DNA content of the established single cell clones remained stable as compared to their parentals, except for JJ012 Cr31-G11 which lost ∼9 chromosomes (Supplementary Fig. S11). WES analysis identified several acquired genetic variants as compared to the parental cell lines, especially in the CH2879 derivates (Supplementary Fig. S10B). However, mock clones showed similar novel variant numbers, suggesting stress or genetic drift rather than off-target CRISPR-Cas9 cleavage as the underlying cause. The variants and affected genes showed minimal overlap between cell lines (Supplementary Fig. S10B-C), indicating a predominantly random pattern, consistent with the over-representation analysis (ORA) results showing no significant pathway enrichment (Supplementary Table S4). All mock and CRISPR-edited single cell clones retained nominal expression of the IDH1 protein and D-2-HG production was present or absent according to their IDH1 mutation status (Fig. 3B-C). No pronounced differences in growth rate were observed between mock and CRISPR-edited clones (Fig. 3D), and thus, JJ012 cells were able to survive without their endogenous IDH1^R132G^ mutation. Hence, a unique panel of CRISPR-edited IDH1^WT^ and IDH1^MUT^ isogenic cell line pairs with balanced IDH1^WT^ and IDH1^MUT^ was established, complementing the previously described vector-based IDH1^MUT^ cell line panel characterized by IDH1^MUT^ overexpression. Moreover, survival of JJ012 cells in which IDH1^MUT^ was reverted to IDH1^WT^ shows the IDH1 mutation does not remain an essential driver in all chondrosarcomas during advanced stages of tumour growth. This provides an explanation for the limited clinical efficacy of IDH1^MUT^ protein inhibitors in a subset of chondrosarcoma patients and underlines the need for the development of alternative therapeutic strategies beyond targeting the IDH mutation.

### CRISPR-edited IDH1^MUT^ chondrosarcoma cell lines do not show synthetic lethal interactions, but addition of exogeneous D-2-HG induces therapeutic vulnerabilities

**Fig. 4:**
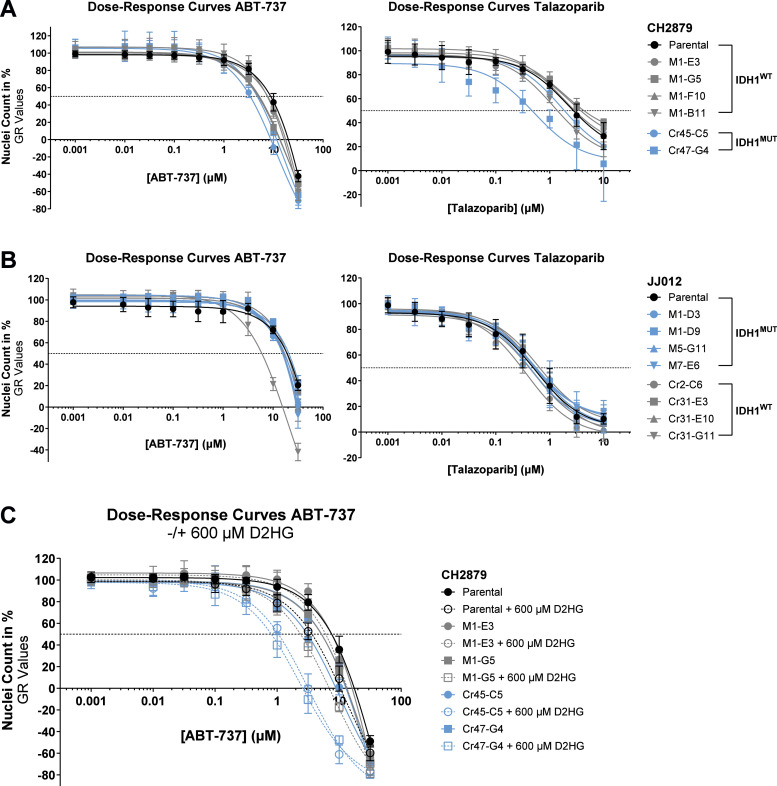
CRISPR-edited IDH1^MUT^ chondrosarcoma cell lines do not show synthetic lethal interactions, but addition of exogeneous D-2-HG induces therapeutic vulnerabilities. Dose-response curves of three days ABT-737 (Bcl-2/Bcl-xL/Bcl-w inhibitor) and talazoparib (PARP inhibitor) treatment in **(A)** CH2879 and **(B)** JJ012 parental, mock, and CRISPR-edited IDH^WT^ and IDH1^MUT^ chondrosarcoma cell lines. Data were corrected for growth rate differences between cell lines (GR values) and GR_50_ values are listed in Table 2. Data points represent the mean ± standard deviation of three independent experiments performed in triplicate. **(C)** Dose-response curves of three days ABT-737 (Bcl-2/Bcl-xL/Bcl-w inhibitor) ± 600 µM D-2-HG treatment in CH2879 parental, mock, and CRISPR-edited IDH^WT^ and IDH1^MUT^ cell lines. Data were corrected for the monotherapy effect of 600 µM D-2-HG and growth rate differences between cell lines (GR values). GR_50_ values, including percentage of reduction between ± 600 µM D-2-HG conditions, are listed in Table 2. Data points represent the mean ± standard deviation of three independent experiments performed in triplicate.

In contrast to the vector-based IDH1^MUT^ cell lines, but in line with endogenous IDH1^MUT^ chondrosarcoma cell lines, no synthetic lethal interactions between the IDH1 mutation and Bcl-2 family member or PARP inhibition were observed in the CRISPR-edited single cell clones (Fig. 4A-B, Table 2). CH2879 Cr47-G4 and JJ012 Cr31-G11 showed a shift in talazoparib or ABT-737 response respectively (∼80% and ∼70% reduction in GR_50_), but this was most likely caused by cell line specific characteristics such as novel acquired mutations in e.g., DNA repair pathways (Supplementary Table S4). As the results from the vector-based CH2879 IDH1^MUT^ cell lines suggested IDH1^MUT^ overexpression influences therapeutic vulnerabilities, this phenotype was mimicked in the CRISPR-edited CH2879 IDH1^MUT^ cell lines by adding exogeneous D-2-HG in a concentration (600 µM) that minimally affected CH2879 cell viability as a monotherapy (Supplementary Fig. 12). Both mock and CRISPR-edited CH2879 IDH^WT^ and IDH1^MUT^ became more sensitive to ABT-737 treatment, although the shift in treatment response was more pronounced in the IDH1^MUT^ cell lines (average reduction in GR_50_ of ∼40% and ∼75% respectively) (Fig. 4C, Table 2). Together, these results indicate that synthetic lethal interactions with the IDH mutation are present or absent depending on the type of preclinical model used, and underlines the significant role IDH1^MUT^ copy number, IDH1^MUT^ protein expression, and/or D-2-HG levels play in shaping these treatment vulnerabilities. Researchers performing future preclinical studies should therefore carefully select their artificially created IDH^MUT^ *in vitro* models and prioritize the use of endogenous IDH^MUT^ models, if available, to avoid potential gaps in the bench-to-bedside translation of promising therapeutic strategies.

**Table 2:** GR_50_ values of CH2879 and JJ012 parental, mock, and IDH1 CRISPR-edited cell lines.

		**ABT-737 GR_50_** µM	**Talazoparib GR_50_** µM
**CH2879**	**Parental**	8.78	2.76
	**M1-E3**	7.59	3.76
	**M1-G5**	5.16	4.32
	**M1-F10**	4.91	2.91
	**M1-B11**	7.49	1.39
	**Cr45-C5**	3.39	1.88
	**Cr47-G4**	4.68	0.472
**JJ012**	**Parental**	19.0	0.497
	**M1-D3**	15.6	0.428
	**M1-D9**	20.7	0.537
	**M5-G11**	17.1	0.563
	**M7-E6**	15.4	0.511
	**Cr2-C6**	15.9	0.303
	**Cr31-E3**	18.9	0.539
	**Cr31-E10**	16.7	0.650
	**Cr31-G11**	5.96	0.419
**CH2879 -/+ 600 µM D-2-HG**	**Parental**	7.58 vs. 3.64 (52% reduction)	N/D
	**M1-E3**	7.58 vs. 6.05 (20% reduction)	N/D
	**M1-G5**	5.16 vs. 2.38 (54% reduction)	N/D
	**Cr45-C5**	3.07 vs. 1.05 (66% reduction)	N/D
	**Cr47-G4**	5.19 vs. 0.805 (84% reduction)	N/D

GR_50_ = growth rate corrected equivalent of the IC_50_ value; N/D = not determined.

## Discussion

Synthetic lethal interactions with the IDH mutation have been identified in preclinical studies, but clinical translation has remained limited. This might be ascribed to the use of artificially created IDH^MUT^ models that poorly reflect endogenous IDH^MUT^ tumour biology. To investigate if the utilized IDH^MUT^ model (endogenous versus genetically modified) is indeed a defining factor, a unique panel of isogenic IDH^WT^ and IDH1^MUT^ chondrosarcoma cell lines was established. Despite differences in doubling times, IDH1^R132C^ copy numbers, IDH1^R132C^ protein expression, and D-2-HG production, most vector-based cell lines showed the previously reported synthetic lethal interactions with Bcl-2 family member inhibitors, PARP inhibitors, and radiotherapy [[19], [20], [21], [22], [23], [24], [25], [26]]. In contrast, these therapeutic vulnerabilities were not observed in IDH1 CRISPR-edited models, nor when comparing endogenous IDH1^WT^ and IDH^MUT^ chondrosarcoma cell lines, or when inhibiting the IDH^MUT^ protein [[27], [28], [29]]. In line with the results from the present study, Garrett *et al.* previously reported reduced glutamine and glutamate levels in an artificially created IDH^MUT^ glioma model, whilst this difference was absent when comparing endogenous models [36]. Hence, artificially created IDH^MUT^ models do not always fully recapitulate the downstream biological effects induced by endogenous IDH mutations, resulting in underlying therapeutic vulnerabilities that may normally not be present in endogenous IDH^MUT^ tumours. The use of such non-representative models could lead to false positive preclinical discoveries, resulting in negative clinical trials with no or limited durable clinical responses. Whilst PARP inhibition was identified as a promising therapeutic strategy in multiple preclinical IDH^MUT^ models ([20,23],[24]), recent clinical trials do not demonstrate clinical benefit in IDH^MUT^ AML, cholangiocarcinoma and chondrosarcoma patients (NCT03953898) ([37],[38]), Additionally, only a subset of IDH^MUT^ glioma patients shows a response or stabilization of the disease ([39],[40]). Future clinical trials should elucidate if the translation of other therapeutic strategies identified in preclinical IDH^MUT^ models is more successful. Nevertheless, researchers performing preclinical studies should be cautious with the use of artificially created IDH^MUT^ models and prioritize the use of endogenous IDH^MUT^ models, if available, to avoid false positive discoveries and reduce discrepancies between preclinical and clinical studies.

Differences in underlying therapeutic vulnerabilities between IDH^MUT^ models might be ascribed to various aspects. First, most artificial IDH^MUT^ models were established by lentiviral transduction, which often leads to IDH^MUT^ overexpression due to a potentially high copy number integration or the use of a strong, non-endogenous promoter (e.g., CMV) [19]. Despite usage of the endogenous IDH1 promoter in the current study, IDH1^MUT^ copy number, IDH1^MUT^ protein expression, and D-2-HG levels were identified as factors contributing to treatment response. This is supported by the finding that treatment with AGI-5198 could not completely abolish D-2-HG production in the vector-based IDH^MUT^ models, resulting in a partial treatment rescue, whereas elevated D-2-HG levels sensitized CRISPR-edited models to treatment. A previous study found that tumours harbouring weak D-2-HG-producing mutations show reduced DNA hypermethylation levels as compared to other IDH variants, indicating the pronounced effect D-2-HG levels have on shaping IDH^MUT^-induced changes [5]. In combination with our finding that certain therapeutic vulnerabilities are absent in endogenous and CRISPR-edited models, which are both characterized by balanced IDH1^MUT^ expression, the current study suggests that overexpression of the IDH mutation and/or high D-2-HG levels are key factors to consider when studying underlying therapeutic vulnerabilities in preclinical models of IDH^MUT^ tumours.

This study also highlights that not all vector-based high IDH^MUT^ copy number cell lines harboured the same underlying vulnerabilities, nor were these treatment sensitivities always retained whilst culturing over time, implying other factors beyond IDH1^MUT^ overexpression and/or high D-2-HG levels contribute to therapy response. The current study shows pronounced biological differences between and within cell lines that go beyond apoptotic and DNA repair signalling pathways. This implies the vector-based IDH^MUT^ cell lines harbour distinct (epi)genetic or transcriptomic backgrounds in which the IDH1 mutation was introduced, likely caused by sub-clonality within the parental cell lines (e.g., different TP53 mutations in the CH2879 cell line) [28], genomic scars caused by lentiviral vector integration, (epi)genetic drift whilst culturing over time, or adaptation to the introduced IDH1 mutation. Recent studies have shown that the (epi)genetic context in which IDH^WT^ and IDH^MUT^ are embedded influences therapy response as well as patient outcome [[41], [42], [43], [44], [45], [46], [47]]. For instance, the mutational statuses of TP53 and ATRX determine whether IDH^WT^ and IDH^MUT^ gliomas respond to radiotherapy [41]. Introduction of an IDH mutation in a generic cancer cell line harbouring several additional mutations could therefore induce underlying therapeutic vulnerabilities that are normally not present in IDH^MUT^ tumours. This underlines the importance of studying the IDH mutation in a relevant, naturally occurring (epi)genetic and transcriptomic background and highlights this aspect as another key factor to consider when modelling IDH^MUT^ tumours.

Additionally, this study indicated that JJ012 cells remained viable and proliferative when their endogenous IDH1^MUT^ was reverted to an IDH1^WT^, implying not all IDH^MUT^ tumours are, or remain, dependent on this mutation for growth. This is in line with previous findings where inhibition of IDH^MUT^ in chondrosarcoma cell lines did not or only moderately decreased cell viability, colony formation and migration ([48],[49]). In addition, only a subset of chondrosarcoma patients, primarily those with few co-occurring mutations, responds to IDH^MUT^ inhibitors ([11],[12]), underlining the need for alternative therapeutic strategies. IDH mutations most likely play a prominent role in early tumourigenesis, as these mutations are even more frequently observed in enchondroma (87%) [50], the benign precursor of chondrosarcoma. Moreover, recent studies report on the loss of IDH mutations in recurrent gliomas and metastasized chondrosarcomas ([51],[52]). Interestingly, some recurrent gliomas gained or amplified their IDH1^MUT^ allele as compared to their corresponding primary tumours. Additional research is needed to elucidate if tumours positively select for or against the IDH mutation during progression or if these changes are random caused by chromosomal instability. Nevertheless, introduction of an IDH mutation in a well-established cancer cell line does not mimic the natural early onset of these mutations nor the potentially acquired independence of this mutation in later stages of tumour development, underlining another important factor to consider when modelling the biological effects of an IDH mutation. To fully capture the biological consequences of an IDH mutation during the different phases of tumour growth, cell-of-origin models should be established rather than introducing IDH mutations in cell lines that are already tumourigenic or using endogenous IDH^MUT^ models representing more advanced tumour stages. The value of such models was already shown in a study by Suijker *et al.*, which described that addition of exogeneous D-2-HG to mesenchymal stem cells, the presumed cell-of-origin of chondrosarcoma, leads to a block in osteogenic differentiation and an increase in differentiation towards the chondrogenic lineage [53].

In summary, this study highlights that the utilized model system is a decisive factor in determining whether synthetic lethal interactions with the IDH mutation are observed. By comparing vector-based IDH^MUT^ overexpression models with balanced IDH^MUT^ CRISPR-edited models and endogenous IDH^MUT^ cell lines, we demonstrate that therapeutic vulnerabilities can be an artifact of the preclinical model rather than an inherent feature of the disease. Given the high costs of clinical trials and increasingly constrained research budgets, improving the translational relevance of preclinical models is essential to avoid pursuing false positive therapeutic discoveries. Therefore, future preclinical research should prioritize the use of endogenous IDH^MUT^ models, if available, to bridge the bench-to-bedside gap and ensure that identified vulnerabilities are clinically relevant.

## Data Availability

The manuscript and supplementary files contain all potential findings based on raw data analysis. The WES and RNA sequencing data were deposited in the European Genome-phenome Archive (EGA) under dataset accession number EGAD50000002703. Other raw data are available from the corresponding author on reasonable request.

## Funding

This work was financially supported by the Dutch Research Council (ZON-MW VICI 170.055 to JVMGB) and the 10.13039/100011719Bone Cancer Research Trust (grant number 8922 to SV & JVMGB).

## CRediT authorship contribution statement

**Alwine B. Kruisselbrink:** Writing – review & editing, Methodology, Investigation, Conceptualization. **Tessa A.H. Wilpshaar:** Writing – review & editing, Methodology, Investigation. **Ieva Palubeckaitė:** Writing – review & editing, Methodology, Investigation, Conceptualization. **Romy C. Dijkland:** Writing – review & editing, Investigation. **Tatiana Belova:** Writing – review & editing, Software, Formal analysis. **Sara Cardoso:** Writing – review & editing, Software, Formal analysis. **Pauline M. Wijers-Koster:** Writing – review & editing, Investigation. **Inge H. Briaire-de Bruijn:** Writing – review & editing, Investigation. **René J.M. van Zeijl:** Writing – review & editing, Investigation. **Hans Dalebout:** Writing – review & editing, Investigation. **Marieke L. Kuijjer:** Writing – review & editing, Supervision, Conceptualization. **Hailiang Mei:** Writing – review & editing, Software, Formal analysis. **Bram Heijs:** Writing – review & editing, Supervision, Conceptualization. **Karoly Szuhai:** Writing – review & editing, Supervision, Conceptualization. **Judith V.M.G. Bovée:** Writing – review & editing, Supervision, Funding acquisition, Conceptualization. **Sanne Venneker:** Writing – review & editing, Writing – original draft, Visualization, Validation, Supervision, Project administration, Methodology, Investigation, Funding acquisition, Formal analysis, Conceptualization.

## Declaration of competing interest

The authors declare that they have no known competing financial interests or personal relationships that could have appeared to influence the work reported in this paper.

## Appendix Supplementary materials

Image, application 1

Image, application 2

Image, application 3
