# Supplementary material for: Targeting vulnerabilities in IDH mutant tumours: The model matters
Source: Neoplasia. 2026 Jul 27;80:101338. doi: 10.1016/j.neo.2026.101338 (PMC13446203; doi:10.1016/j.neo.2026.101338)
Supplement: Supplementary file 1 [file mmc1.pdf]

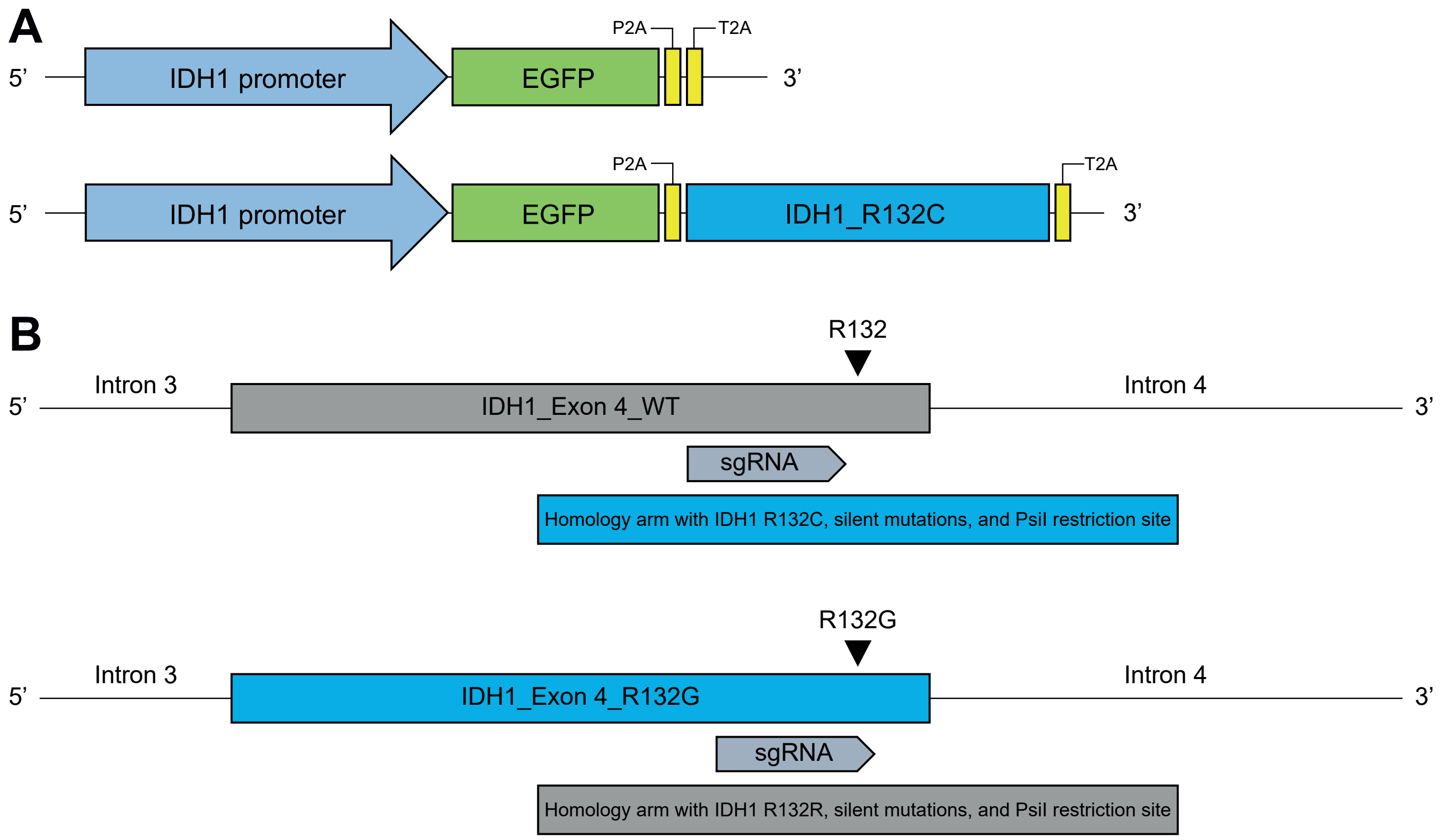

Supplementary Figure 1. Schematic overviews of the lentiviral vectors and CRISPR-Cas9 gene editing strategies used in this study.

(A) Schematic overview of the EV and the IDH1 p.R132C variant lentiviral vectors. Both vectors have the pUltra vector (Addgene plasmid # 24129) as backbone and contain the endogenous IDH1 promoter to initiate expression of EGFP alone or bicistronic expression of EGFP and IDH1^{MUT}. P2A/T2A sites between EGFP and IDH1^{MUT} enable translation of individual proteins from one RNA transcript. **(B)** Schematic overview of the CRISPR-Cas9 homology directed gene editing strategy to introduce the IDH1 p.R132C mutation or revert an endogenous IDH1 p.R132G mutation to an IDH1 WT allele. sgRNAs were designed around the R132 target site and homology arms of 150 bp harboured the desired IDH1 point mutation, silent mutations in the PAM region to prevent additional Cas9 cleavage after successful editing, and a novel PsiI restriction site to verify if homology directed repair occurred. DNA sequences of sgRNAs and homology arms are listed in Supplementary Table S1.

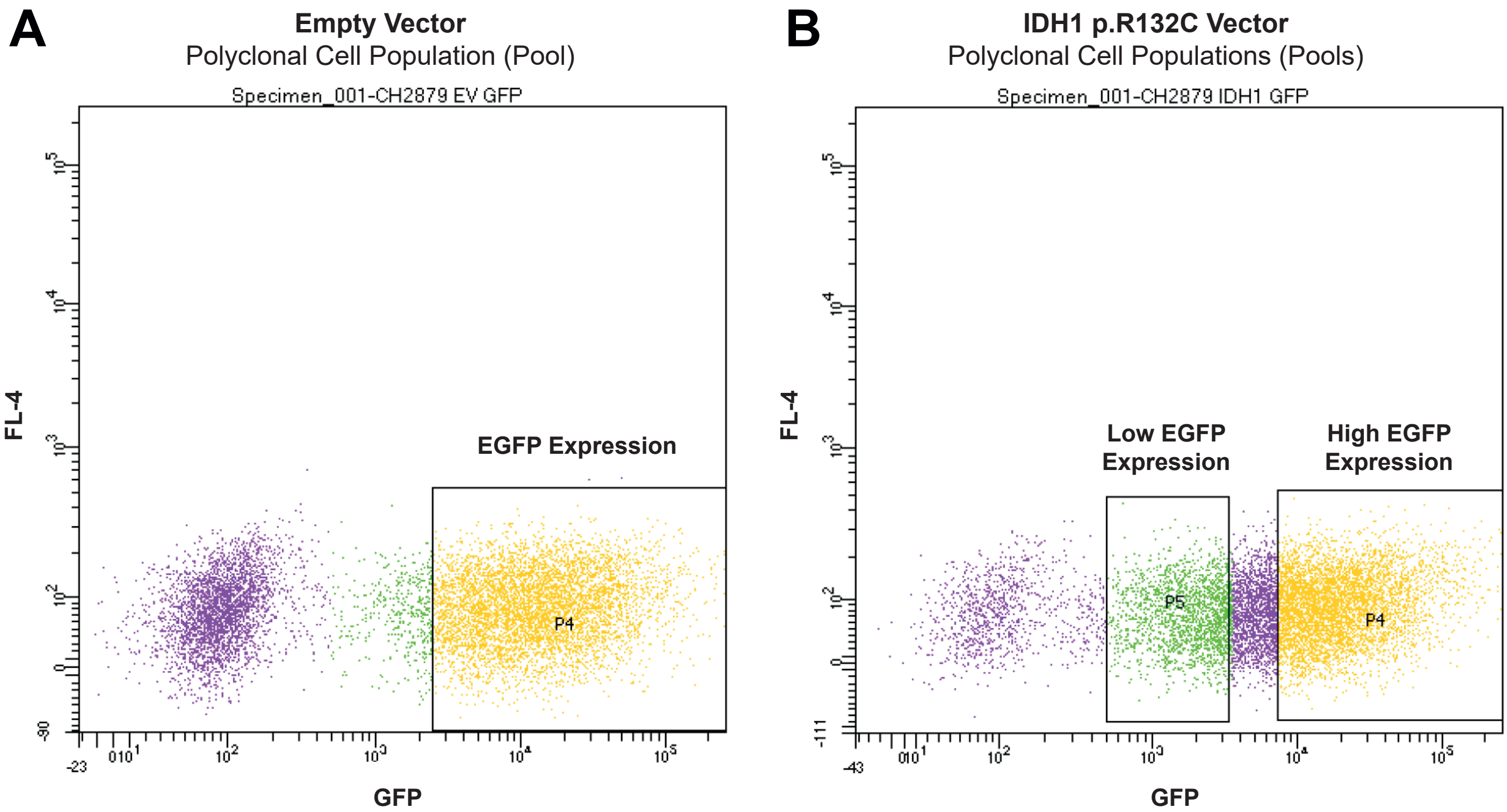

Supplementary Figure 2. Establishment of vector-based CH2879 EV and $IDH1^{MUT}$ polyclonal cell populations. Transduced CH2879 cells were sorted on EGFP expression by FACS, resulting in **(A)** the empty vector polyclonal cell population with mixed EGFP expression (P4 gate) and **(B)** $IDH1^{MUT}$ polyclonal cell populations with relatively low (P5 gate) and high (P4 gate) EGFP expression, representing a low and a high copy number of the IDH1 p.R132C variant, respectively.

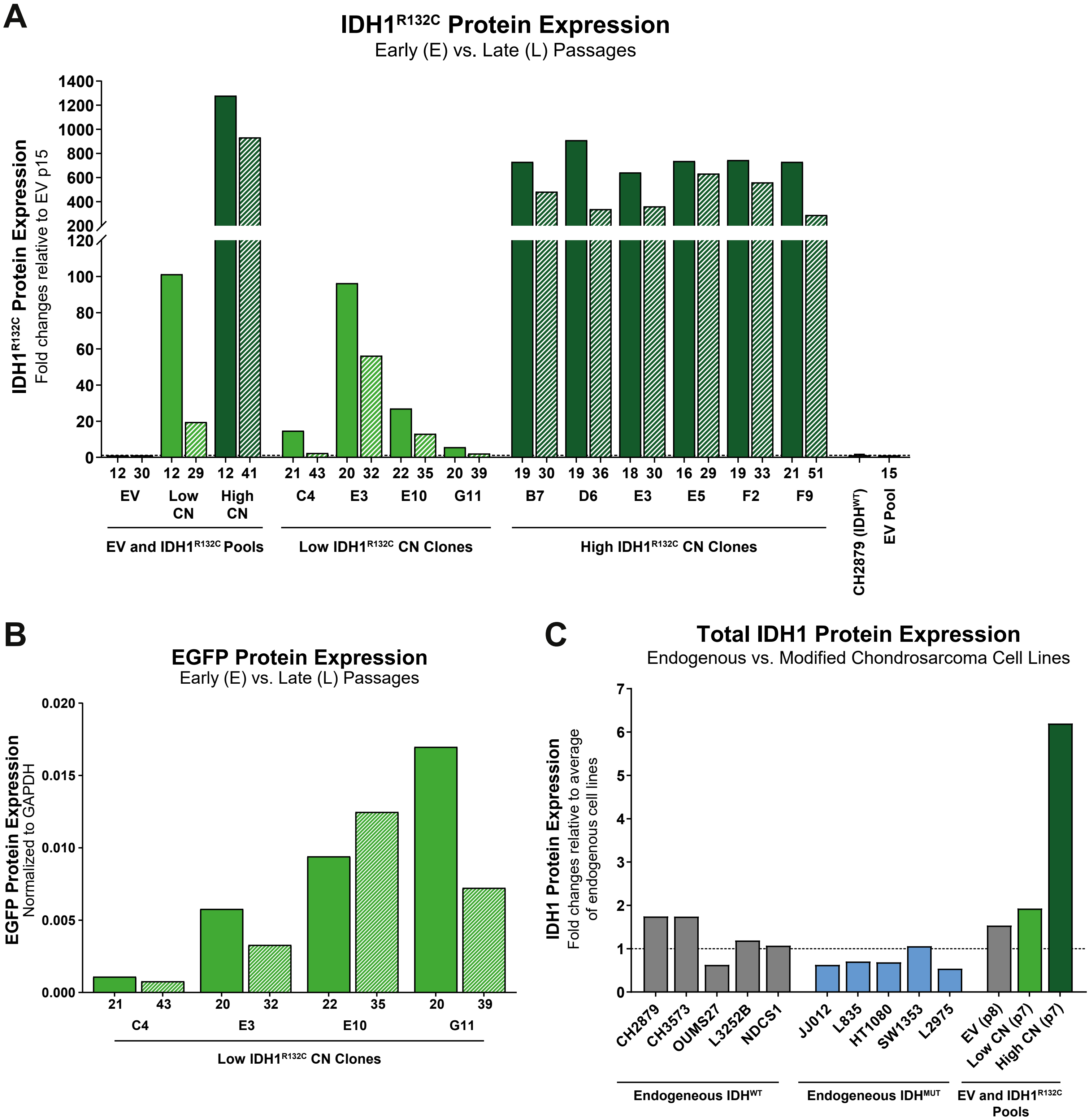

Supplementary Figure 3. Quantification of IDH1^{R132C}, EGFP, and total IDH1 protein expression. **(A)** Quantification of IDH1^{R132C} protein expression levels. Raw data were normalized to GAPDH expression levels and fold changes were calculated relative to EV p15. Passage numbers indicate the number of passages after lentiviral transduction. A Kruskal–Wallis test followed by a Dunn's post-hoc test did not detect statistically significant changes ($p \leq 0.05$) between early and late passages of individual cell lines. **(B)** Quantification of EGFP protein expression levels in low IDH1^{R132C} CN clones. Raw data were normalized to GAPDH expression levels. Passage numbers indicate the number of passages after lentiviral transduction. **(C)** Quantification of total IDH1 protein expression levels. Raw data were normalized to GAPDH expression levels and fold changes were calculated relative to the average expression levels of endogenous IDH^{WT} and IDH^{MUT} chondrosarcoma cell lines. Passage numbers indicate the number of passages after lentiviral transduction.

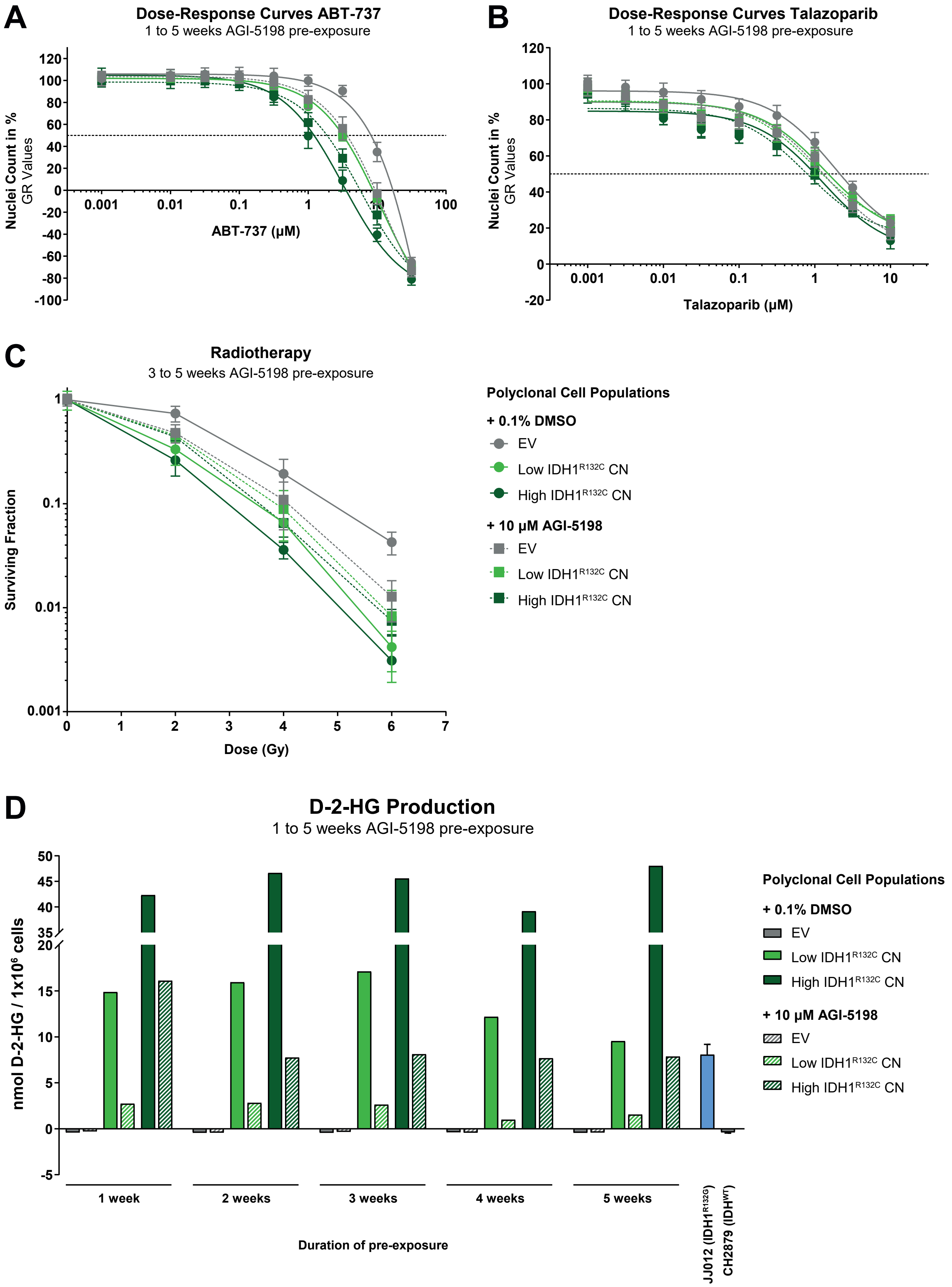

Supplementary Figure 4. Exposure to the IDH1^{MUT} protein inhibitor AGI-5198 cannot completely abolish therapy response nor D-2-HG production in vector-based CH2879 IDH1^{MUT} cell lines.
Dose-response curves of three days **(A)** ABT-737 (Bcl-2/Bcl-xL/Bcl-w inhibitor) and **(B)** talazoparib (PARP inhibitor) ± AGI-5198 (IDH1^{MUT} protein inhibitor) treatment. Polyclonal CH2879 EV and IDH1^{MUT} cell populations were pre-exposed to 0.1% DMSO or 10 µM AGI-5198 for one to five weeks. Data were corrected for growth rate differences between cell lines (GR values). Data points represent the mean ± standard deviation of four independent experiments performed in triplicate. **(C)** Surviving fractions after treatment with single dosages of γ-radiation ± AGI-5198 (IDH1^{MUT} protein inhibitor) in polyclonal CH2879 EV and IDH1^{MUT} cell populations pre-exposed to 0.1% DMSO or 10 µM AGI-5198 for one to five weeks. Data points represent the mean ± standard deviation of three independent experiments performed in duplicate. **(D)** D-2-HG production per one million cells measured by LC-MS/MS. CH2879 and JJ012 cells were included as positive and negative controls, respectively. Bars represent individual samples that were measured one time.

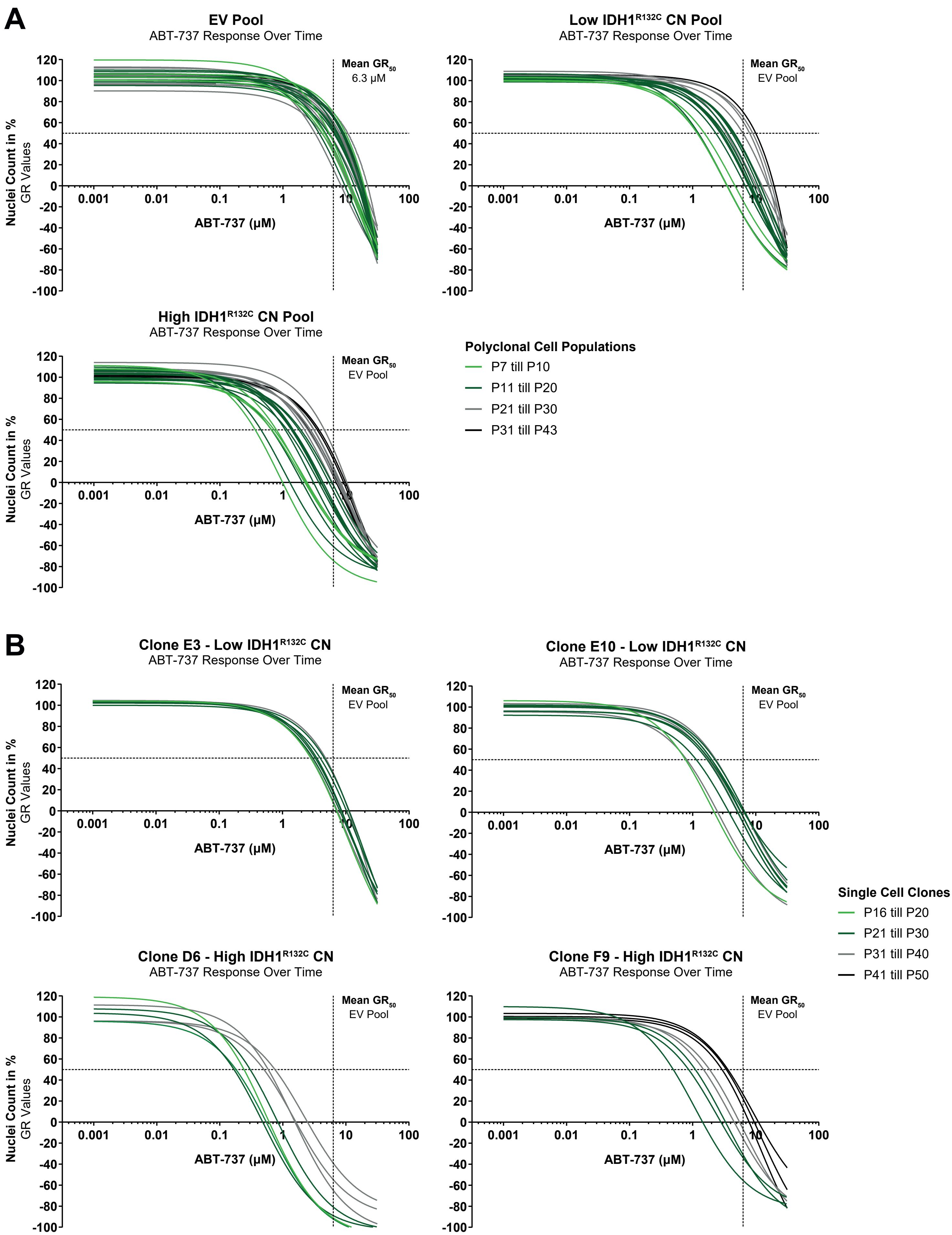

Supplementary Figure 5. Some vector-based CH2879 IDH1^{MUT} chondrosarcoma cell lines acquire ABT-737 therapy resistance whilst culturing over time.

Dose-response curves of three days ABT-737 (Bcl-2/Bcl-xL/Bcl-w inhibitor) treatment in different passage numbers of vector-based CH2879 EV and IDH1^{MUT} **(A)** polyclonal cell populations and **(B)** single cell clones. Dose-response curves were corrected for growth rate differences between cell lines (GR values). Nonlinear curve fits represent the mean of one individual experiment performed in triplicate. Experiments were performed over the course of two years by two independent researchers.

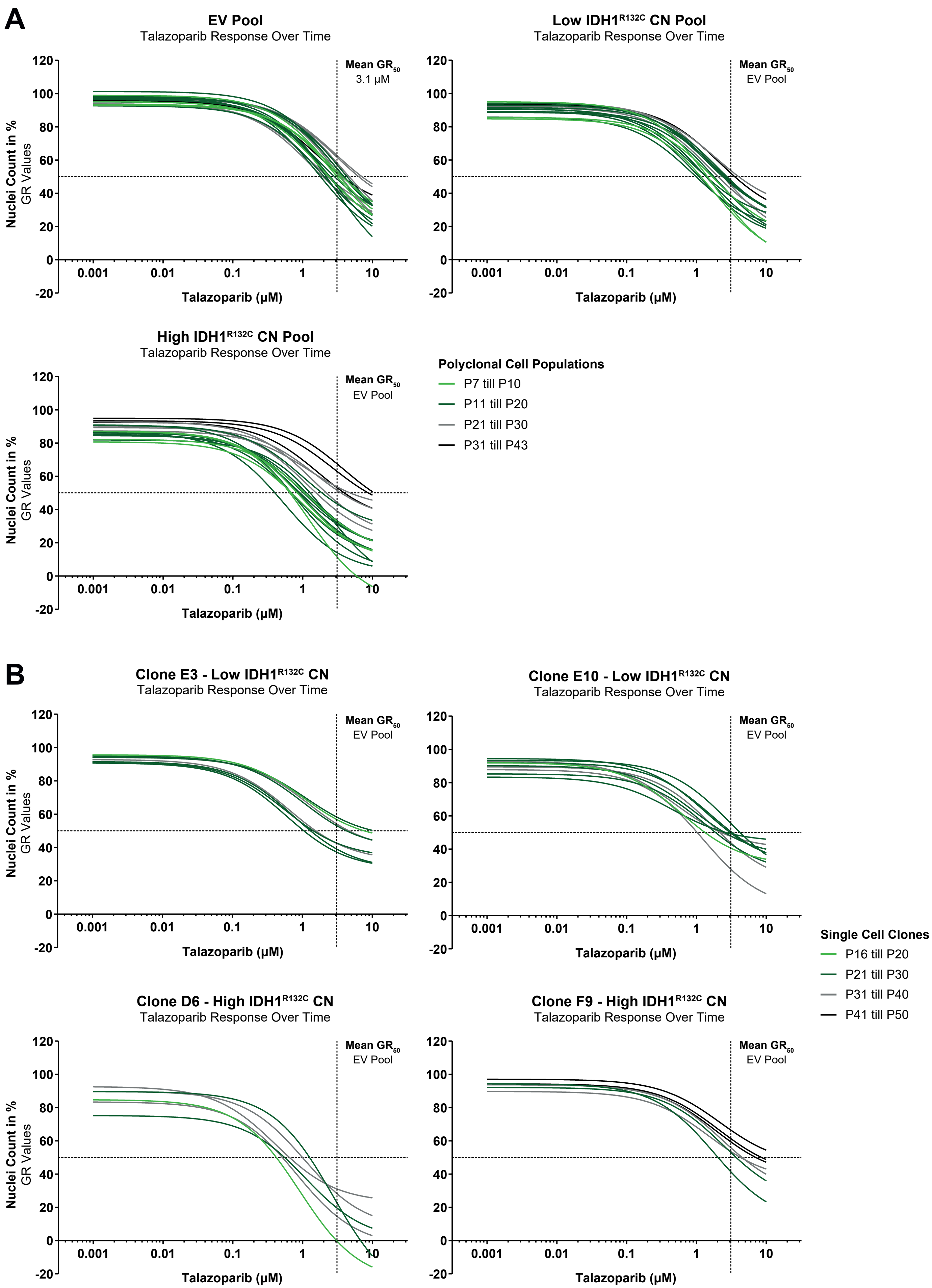

Supplementary Figure 6. The CH2879 high IDH1^{MUT} CN polyclonal cell population acquires talazoparib resistance whilst culturing over time.

Dose-response curves of three days talazoparib (PARP inhibitor) treatment in different passage numbers of vector-based CH2879 EV and IDH1^{MUT} **(A)** polyclonal cell populations and **(B)** single cell clones. Dose-response curves were corrected for growth rate differences between cell lines (GR values). Nonlinear curve fits represent the mean of one individual experiment performed in triplicate. Experiments were performed over the course of two years by two independent researchers.

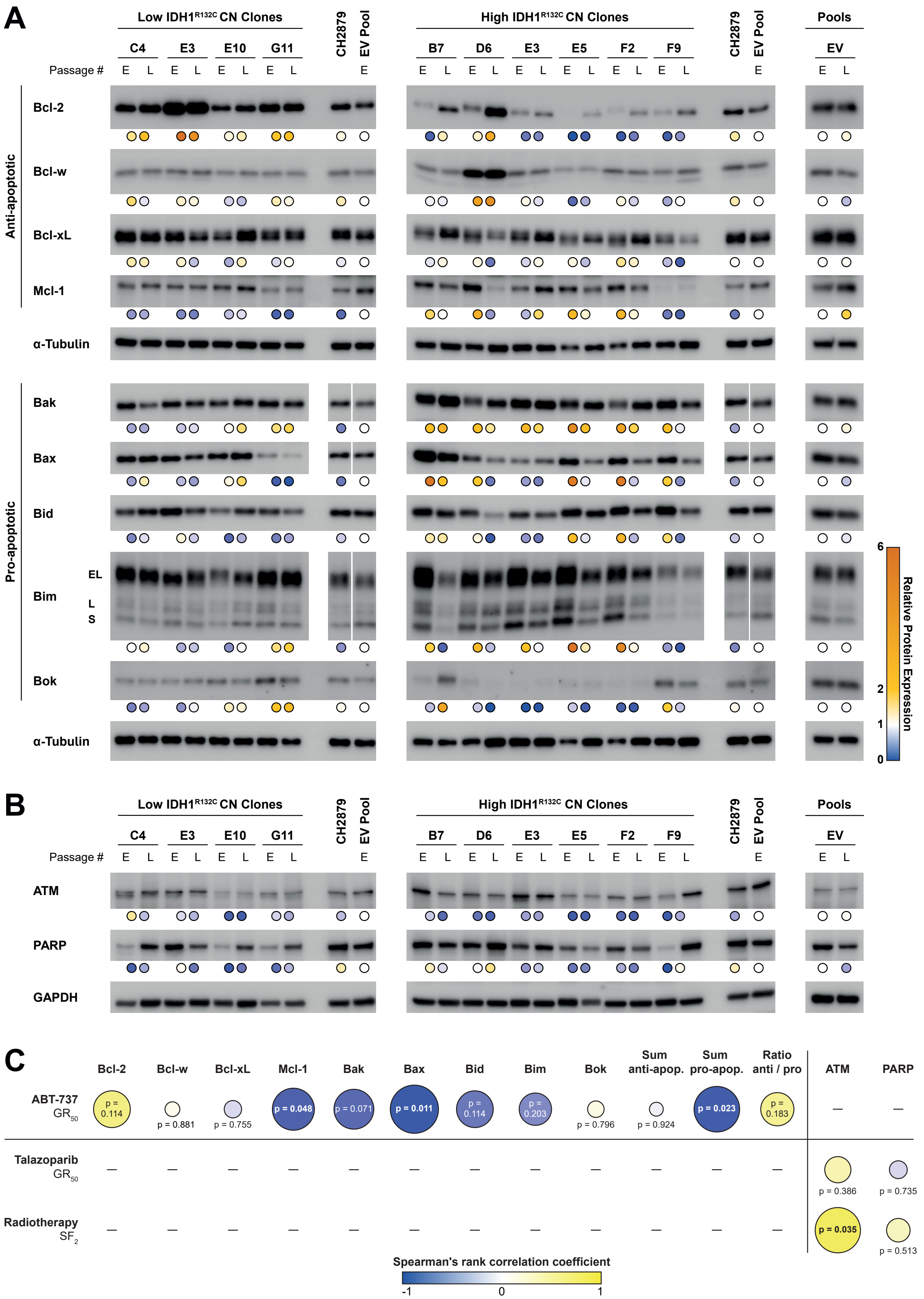

Supplementary Figure 7. Proteins related to apoptosis and DNA repair are differentially expressed between vector-based IDH1^{MUT} chondrosarcoma cell lines.

Expression of **(A)** anti- and pro-apoptotic Bcl-2 family member proteins and **(B)** DNA repair proteins in early (E) and late (L) passages of vector-based CH2879 IDH1^{MUT} cell lines. Passage numbers are comparable to those depicted in Figure 1A. CH2879 parental and EV early passage cells were included as negative controls on each western blot. Samples were rearranged for visualisation. α-Tubulin expression was used as a loading control. Raw expression levels were normalized to α-tubulin expression and EV early passage to obtain fold changes, which are represented in the heatmap below the blots. Heatmap colours indicate increased (yellow/orange), stable (white), or reduced (blue) expression as compared to the early passage of the EV pool. **(C)** Spearman correlations of treatment response (GR_{50} and SF_2 values) versus expression of individual proteins. Heatmap colours and circle size represent the spearman's rank correlation coefficients, ranging from -1 (blue) to 1 (yellow) with larger circles representing a stronger positive or negative correlation. Obtained p-values are listed in the circles of the heatmap.

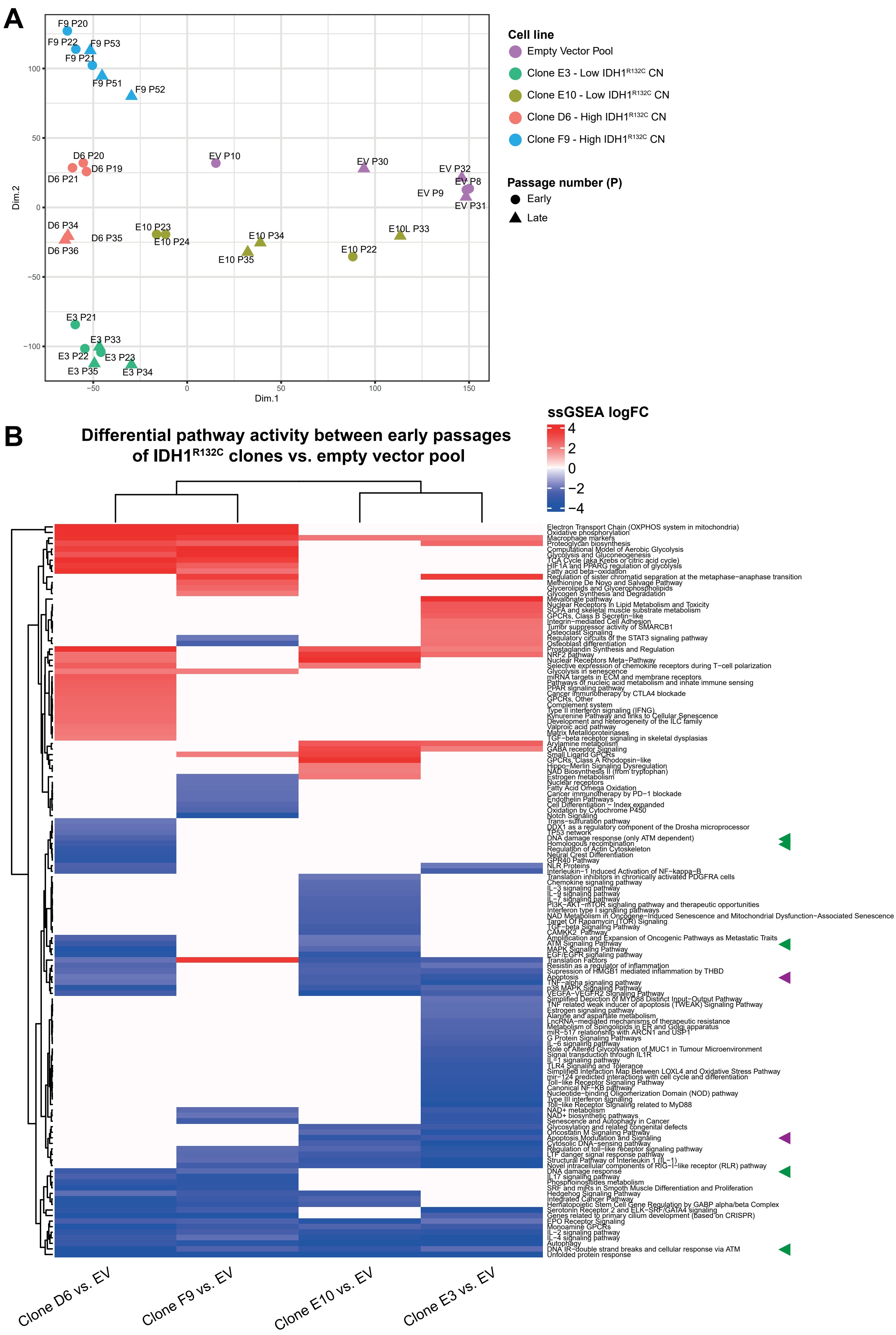

Supplementary Figure 8. Biological differences between vector-based CH2879 IDH1[MUT] chondrosarcoma cell lines. **(A)** Principal component analysis visualizing clustering of samples based on gene expression profiles. **(B)** Single-sample gene set enrichment analysis between early passages of CH2879 EV polyclonal cell population and IDH1[MUT] cell lines. The average pathway activity value of n=3 biological replicates per cell line is depicted in the heatmap as log-fold changes. Thresholds for inclusion were set at log-fold change ≥ 2 and p-adjusted ≤ 0.05. Heatmap colours represent increased (red), stable (white), or reduced (blue) pathway activity as compared to the early passage of the EV pool. Biological pathways involved in apoptosis or DNA repair are indicated with purple and green arrows, respectively.

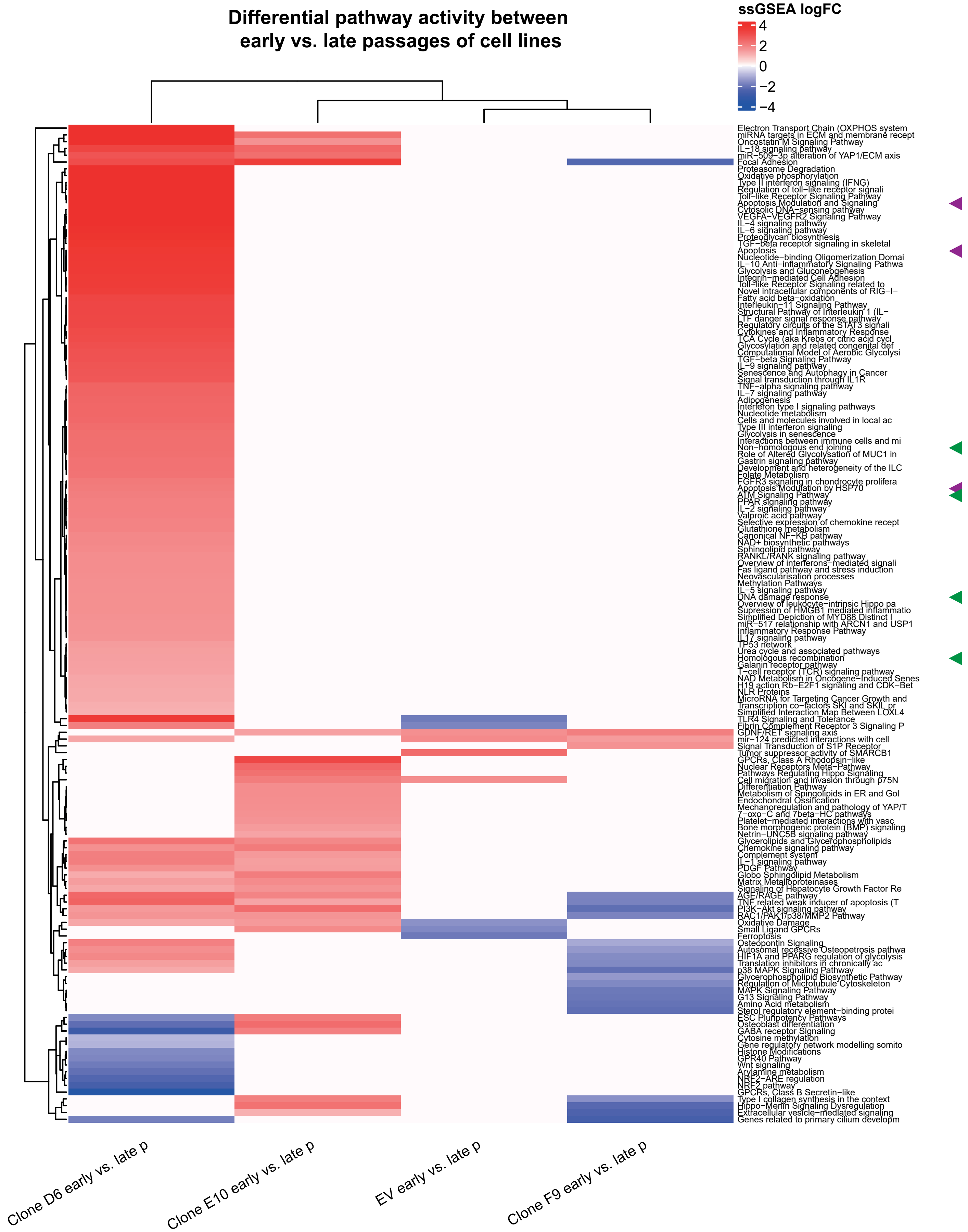

Supplementary Figure 9. Biological differences within vector-based CH2879 IDH1^{MUT} chondrosarcoma cell lines whilst culturing over time.

Single-sample gene set enrichment analysis between early and late passages of individual CH2879 EV and IDH1^{MUT} cell lines. The average pathway activity value of n=3 biological replicates per cell line is depicted in the heatmap as log-fold changes. Thresholds for inclusion were set at p-adjusted ≤ 0.1. No biological differences were observed between early and late passages of clone E3. Heatmap colours represent increased (red), stable (white), or reduced (blue) pathway activity as compared to the early passage of the EV pool. Biological pathways involved in apoptosis or DNA repair are indicated with purple and green arrows, respectively.

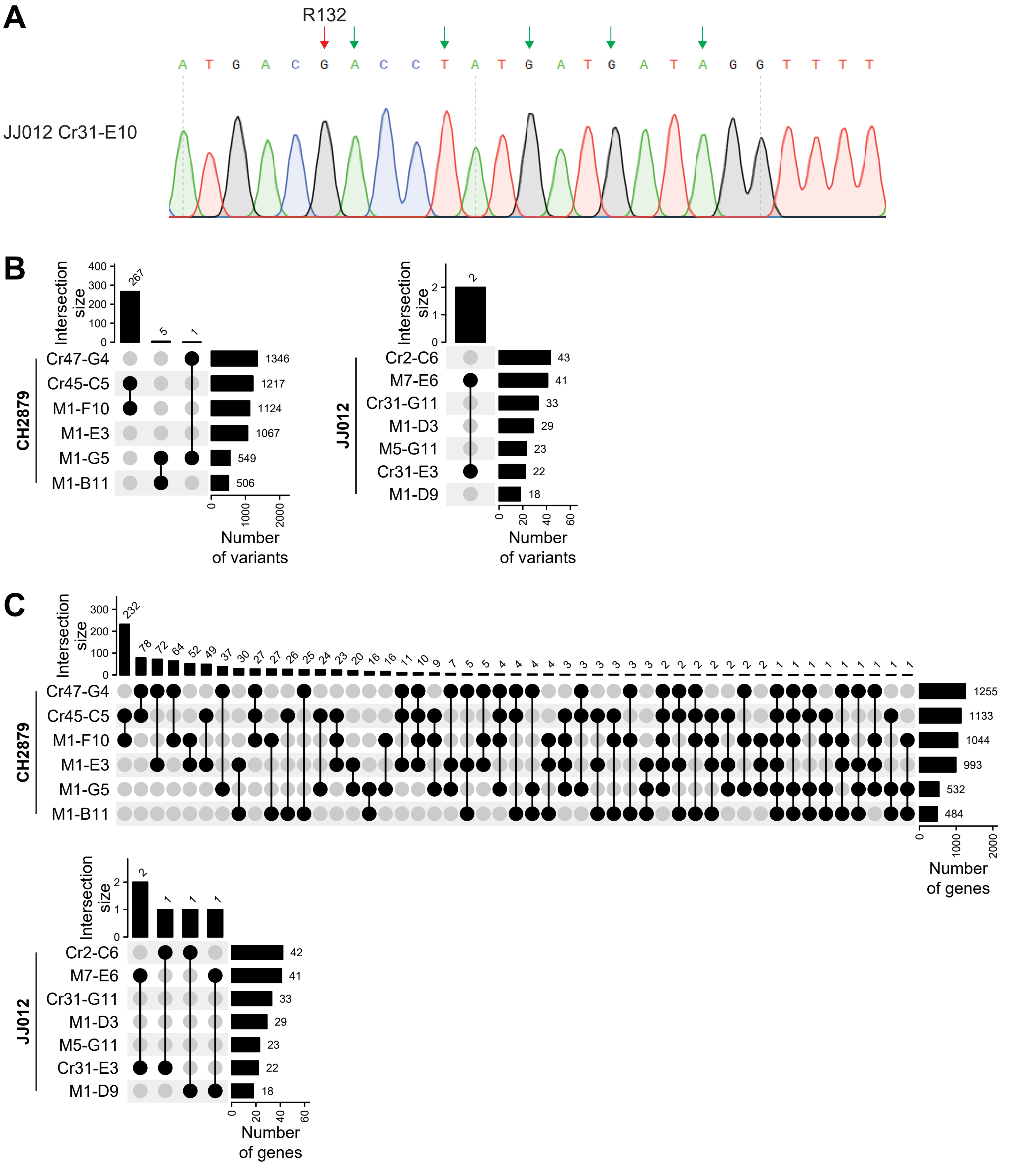

Supplementary Figure 10. Mutational status of mock and CRISPR-edited CH2879 and JJ012 IDH^{WT} and IDH1^{MUT} cell lines. **(A)** Sanger sequencing reads of JJ012 Cr31-E10 cells around the IDH1 R132 CRISPR-Cas9 target site. The red arrow indicates the R132 target site and the green arrows indicate the expected introduced silent mutations after homology directed repair. JJ012 Cr31-E10 cells used their sister chromatid to repair the IDH1 p.R132G mutation, resulting in a lack of the expected silent mutations. **(B & C)** Upset plots for the intersection of **(B)** variants and **(C)** affected genes between mock and CRISPR-edited CH2879 and JJ012 IDH^{WT} and IDH1^{MUT} chondrosarcoma cell lines.

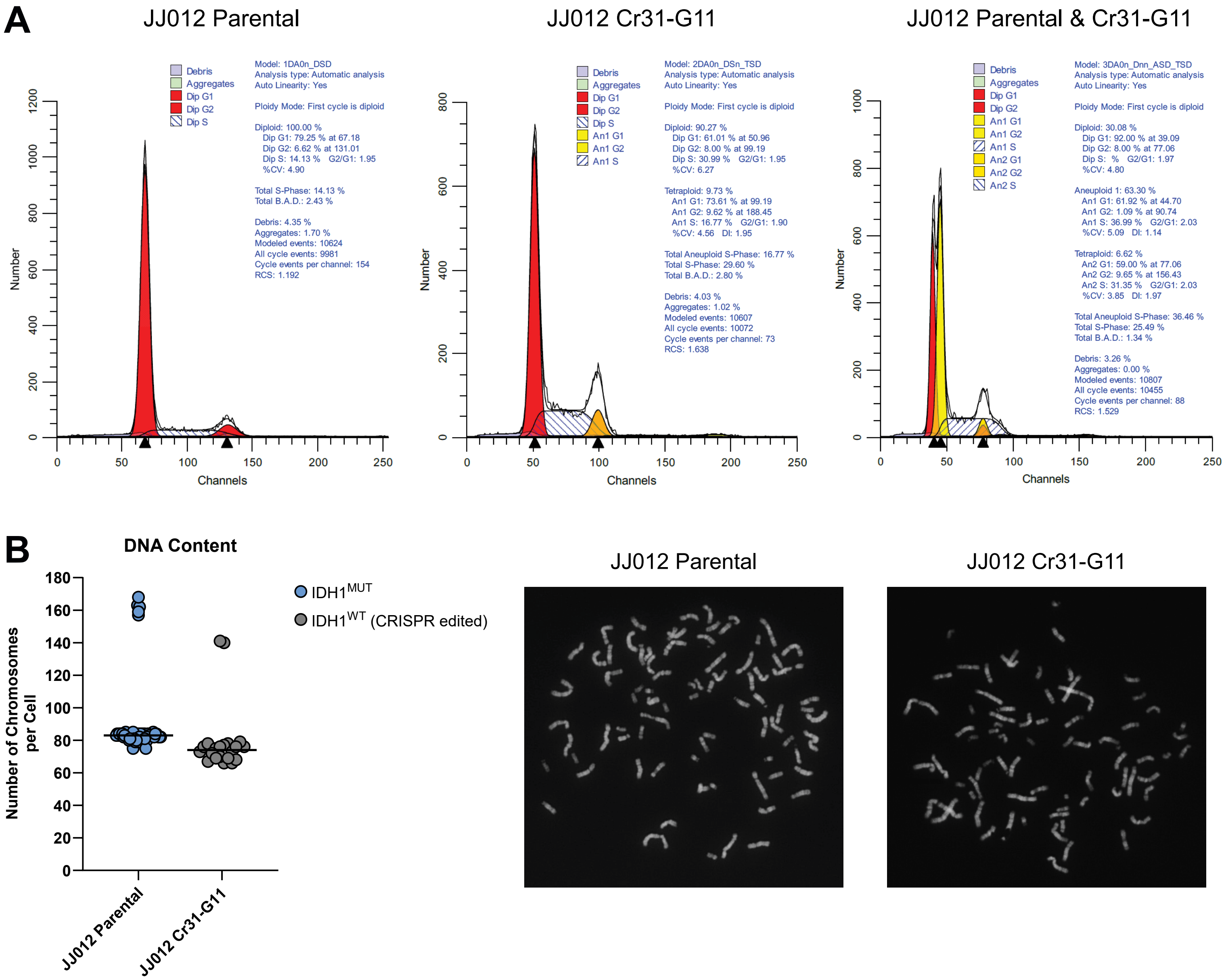

Supplementary Figure 11. Loss of a number of chromosomes in JJ012 Cr31-G11 cells.
(A) DNA histograms of JJ012 parental and Cr31-G11 cells alone or combined. **(B)** Metaphase spreads of JJ012 parental and Cr31-G11 cells. The dot plot depicts the median number of counted chromosomes per metaphase spread, in which every dot represents an individual count (n = >20 per cell line). Some cells underwent endoreduplication and showed a doubled count of the number of chromosomes.

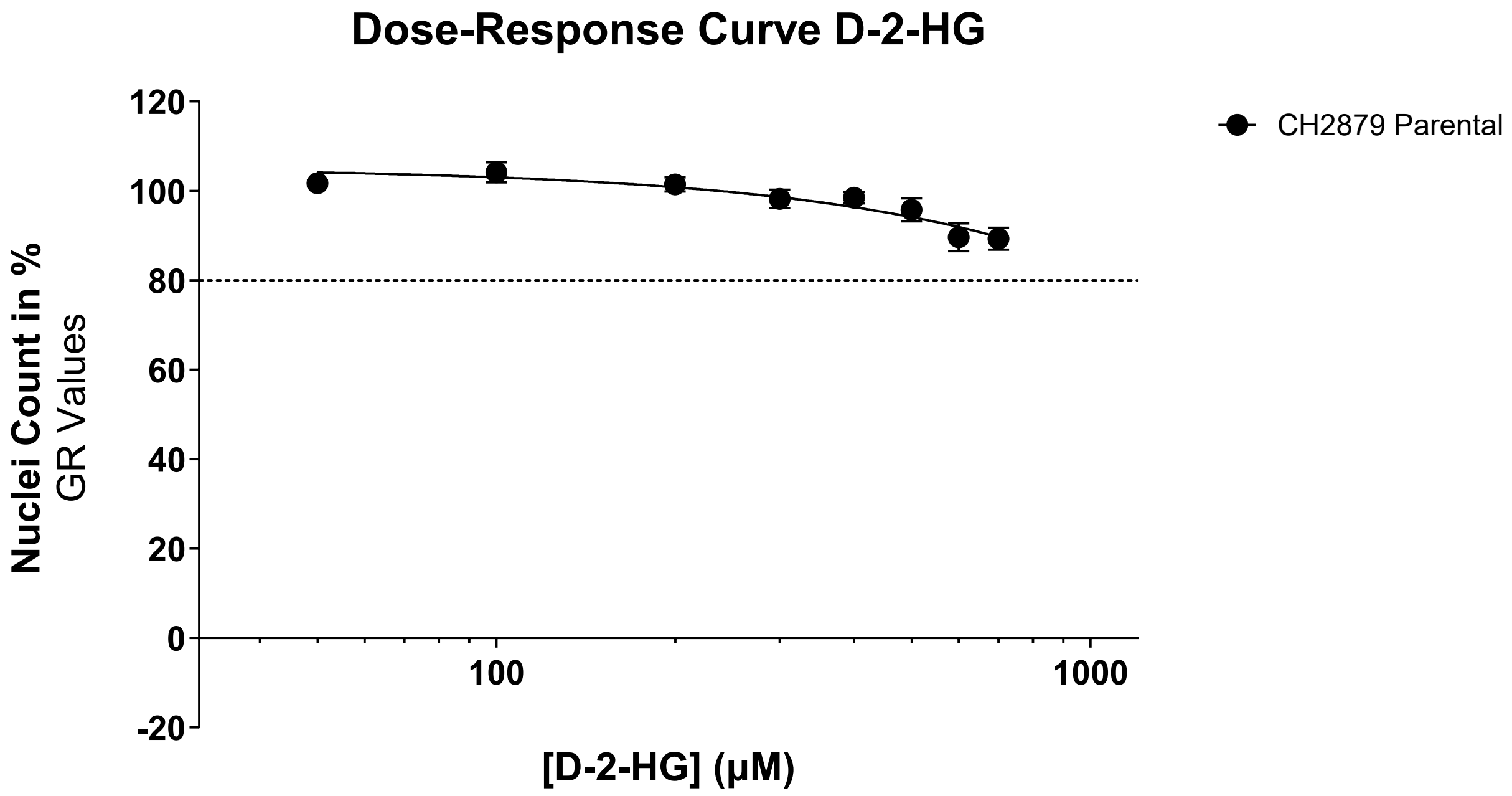

Supplementary Figure 12. Treatment with exogeneous D-2-HG minimally affects the viability of CH2879 cells. Dose-response curves of three days D-2-HG treatment in CH2879 parental cells. The dose-response curve was corrected for growth rate effects (GR values). Data points represent the mean ± standard deviation of one independent experiment performed in triplicate.
