# Supplementary material for: Targeting vulnerabilities in IDH mutant tumours: The model matters
Source: Neoplasia. 2026 Jul 27;80:101338. doi: 10.1016/j.neo.2026.101338 (PMC13446203; doi:10.1016/j.neo.2026.101338)
Supplement: Supplementary file 2 [file mmc2.pdf]

Supplementary Material and Methods

Cloning of empty vector and IDH1^{R132C} vector

The IDH1^{WT} promoter was isolated from DNA from the CH2879 cell line and the IDH1^{R132C} gene was isolated from the pDONR223_IDH1_p.R132C vector, which was a gift from Jesse Boehm, William Hahn and David Root (Addgene plasmid # 81726). Primer pairs to amplify the IDH1^{WT} promoter and the IDH1^{R132C} gene included restriction sites (PacI and AgeI or XbaI and BamHI, respectively) to enable restriction cloning of the PCR products into the pUltra vector, which was a gift from Malcolm Moore (Addgene plasmid # 24129), for bi-cistronic expression of EGFP and IDH1^{R132C} under the endogenous IDH1^{WT} promoter (Supplementary Figure S1A). Polymerase chain reactions (PCRs) were performed with CloneAmp HiFi PCR Premix (Takara Bio, Kusatsu, Shiga, Japan) and the primers listed in Supplementary Table S5 using a Thermal Cycler (Bio-Rad Laboratories, Hercules, CA, USA). The vector and PCR fragments were digested with the corresponding restriction enzymes (New England Biolabs, Ipswich, MA, USA) and the linearized vector was subsequently treated with shrimp alkaline phosphatase (SAP, Takara Bio) for 1 h at 37 °C followed by heat-inactivation at 65 °C for 15 minutes. Digested products were size separated, extracted from the agarose gel, and purified with the NucleoSpin Gel & PCR Clean-up kit (Machery-Nagel, Dueren, Germany) according to the manufacturer's protocol. T4 DNA Ligase (New England Biolabs) was used to clone fragments into the pUltra vector. Stellar Competent Cells (Takara Bio) were transformed with the ligated products and bacteria were spread on LB plates containing 100 µg/mL ampicillin. Plasmids were isolated by mini prep with the NucleoSpin Plasmid mini kit (Machery-Nagel) according to the manufacturer's protocol. Inserts in the vector were verified with Sanger sequencing (see below for detailed methods) and the primers listed in Supplementary Table S5.

Lentivirus production

HEK293T cells were seeded, allowed to attach overnight, and transiently transfected with the pUltra-IDH1^{WT} promoter-IDH1^{R132C} gene vector or the corresponding empty vector control (pUltra-IDH1^{WT} promoter), lentiviral envelope and packaging plasmids (pCMV-VSVG, pRSV-REV, and pMDLg-RRE (gag/pol)), and polyethylenimine (PEI) 25K (23966, Polysciences, Warrington, PA, USA). After overnight incubation, medium was refreshed, and viral supernatants were collected, centrifuged, and filtered (0.45 µm) the next day.

Cell cycle analysis

Cells were trypsinized, counted, and two samples of 1×10^6 cells were prepared per cell line of interest. For each cell line, one of the prepared samples was spiked with an internal standard (i.e., 0.25×10^6 cells of the corresponding parental cell line) to detect potential shifts in DNA content. Cells were fixed, permeabilized, washed, and stained with DAPI as previously described (1). The NucleoCounter NC-250 (Chemometec, Allerod, Denmark) was used to perform cell cycle analysis and data analysis was performed using Winlist 3D and Modfit (Verity Software House, Topsham, ME, USA) as previously described using the automatic analysis setting (1).

Karyotyping

Cells were seeded in 6 well plates (300,000 cells/well) and, after overnight attachment, karyotyping samples were prepared as previously described with 20 minutes of 80 nM Calyculin A treatment (2). Glass slides were mounted and stained using VECTASHIELD

Antifade Mounting Medium with DAPI (Vector Laboratories, Newark, CA, USA) and scanned using a Pannoramic 250 Flash III scanner (3DHistech, Cheshire, UK). Metaphase spreads were annotated and exported as 8-bit TIFF images using QuPath open-source software (https://qupath.github.io/). An ImageJ macro was developed in-house to automatically quantify the number of chromosomes per cell. Briefly, chromosomes were masked using the Phansalkar method with a radius of 5, followed by automatic counting of particles between 50 and 5000 pixels. Images were visually inspected to correct chromosome counts. For each cell line, >20 metaphase spreads were counted.

Sanger sequencing
DNA was isolated using the Wizard Genomic DNA Purification Kit (Promega, Madison, WI, USA) according to the manufacturer's protocol. PCR amplification, purification, and Sanger sequencing on the ABI 3730xl system (Applied Biosystems, ThermoFisher Scientific, Waltham, MA, USA) were performed as previously described with the primers listed in Supplementary Table S5 (3).

Whole exome sequencing and data analysis
DNA was isolated using the Wizard Genomic DNA Purification Kit (Promega) according to the manufacturer's protocol. Whole exome sequencing (WES) using 150bp paired-end reads with a coverage of 100x was performed by Macrogen (Amsterdam, the Netherlands) for parental, mock, and IDH1 CRISPR-edited CH2879 and JJ012 cell lines. Sequencing was performed using the SureSelect Human All Exon V8 library (Agilent Technologies, Santa Clara, CA, USA) on the NovaSeq X platform (Illumina, San Diego, CA, USA). The raw data were deposited in the European Genome-phenome Archive (EGA) under dataset accession number EGAD50000002703.
Raw WES data were processed using the somatic variant calling workflow from BioWDL's germline-DNA pipeline version 5.0.0 (https://biowdl.github.io/) developed by the Sequencing Analysis Support Core (SASC) team at LUMC. Quality control was first performed by FastQC (v0.12.1) and MultiQC (v1.9), followed by adapter clipping using Cutadapt (v4.4). Reads were aligned to the human reference genome GRCh38 using BWAkit (v.0.7.17.dev1). According to the GATK best practice, duplicated mapped reads were marked using Picard (v.3.3.0) and the base quality recalibration was performed using GATK4 (v.4.1.8.0) to generate the analysis-ready reads in the BAM format. VarDict (v1.8.3), Strelka (v2.9.7), and Mutect2 (v4.1.8.0) were used to detect somatic variants in the mock and IDH1 CRISPR-edited CH2879 and JJ012 cell lines, using the respective parental cell line as control to obtain the novel acquired variants for each individual single cell clone. Only variants detected by all variant callers, with ≥1 forward and reverse read containing the variant, and a variant allele frequency (VAF) of at least 10%, as means to correct for genetic drift during culturing, were included in the analysis. Variants also present in the respective parental cell line in ≥1 forward and reverse read were excluded as well, as means to correct for outgrowth of subclones present within the parentals. All variants from JJ012 cell lines that passed these conditions were manually checked using IGV. Due to the high number of detected variants in the CH2879 cell lines, only a subset was manually checked. The variants selected for manual check followed one of the following requirements: a ClinVar annotation, a VAF of 10-11% or >60%, large percentage of reads with the variant close to the end, only 1 read in forward or reverse, predicted to affect a start or stop codon, and total number of reads covered <20.
UpSet plots were made using the R package ComplexHeatmap (v2.22.0) to assess overlapping variants and affected genes between cell lines derived from the same parental

cell line. Over-representation analysis (ORA) was performed using the R package clusterProfiler (v4.14.6) and an in-house curated WikiPathways GMT file comprising biologically relevant pathways for sarcoma to assess the biological impact of the novel acquired variants in CH2879 and JJ012 IDH^{WT} and $IDH1^{MUT}$ cell lines. Biological pathways with a Benjamini–Hochberg false discovery rate–adjusted p-value of <0.05 were considered significantly over-represented.

RNA sequencing and data analysis

RNA was isolated using the RNeasy kit (Qiagen, Venlo, the Netherlands) combined with DNase treatment according to the manufacturer's protocol. Total RNA sequencing (polyA enriched) using 150bp paired-end reads was performed by GenomeScan (Leiden, The Netherlands) for early and late passages of vector-based CH2879 EV and $IDH1^{R132C}$ cell lines in triplicate. Sequencing was performed using the NEBNext Ultra II Directional RNA Library Prep Kit (New England Biolabs) on the NovaSeq6000 platform (Illumina). The raw data were deposited in EGA under dataset accession number EGAD50000002703.

Raw RNA-seq data were processed using the RNA-seq BioWDL pipeline version 5.0.0 (https://biowdl.github.io/) developed by the SASC team at LUMC as previously described (4). Gene counts were normalised with the "median of ratios" method in the DESeq2 package version 1.36.0 in R and normalised expression values were log2-transformed. A principal component analysis (PCA) plot was generated to visualise sample clustering based on gene expression profiles for all samples.

Normalized expression data were used as input for single-sample gene set enrichment analysis (ssGSEA). Gene sets were obtained from an in-house curated WikiPathways GMT file comprising biologically relevant pathways for sarcoma. ssGSEA scores were calculated using the GSVA R package (v1.46.0) with z-score normalization and a minimum gene set size of five genes, resulting in a pathway-by-sample enrichment matrix. Differential pathway activity between experimental conditions was evaluated using linear modeling implemented in the limma R package (v3.54.2). Pairwise contrasts were defined for early passages of the EV pool versus each $IDH1^{R132C}$ clone as well as between early and late passages of each cell line. For each contrast, moderated t-statistics, log-fold changes, and associated p-values were computed, and statistical significance was determined using a Benjamini–Hochberg false discovery rate–adjusted p-value.

References

1. Venneker S, Kruisselbrink AB, Briaire-de Bruijn IH, de Jong Y, van Wijnen AJ, Danen EHJ, et al. Inhibition of PARP Sensitizes Chondrosarcoma Cell Lines to Chemo- and Radiotherapy Irrespective of the IDH1 or IDH2 Mutation Status. Cancers (Basel). 2019;11(12).
2. Franceschini N, Verbruggen B, Tryfonidou MA, Kruisselbrink AB, Baelde H, de Visser KE, et al. Transformed Canine and Murine Mesenchymal Stem Cells as a Model for Sarcoma with Complex Genomics. Cancers (Basel). 2021;13(5).
3. Venneker S, van Eenige R, Kruisselbrink AB, Palubeckaite I, Taliento AE, Briaire-de Bruijn IH, et al. Histone Deacetylase Inhibitors as a Therapeutic Strategy to Eliminate Neoplastic "Stromal" Cells from Giant Cell Tumors of Bone. Cancers (Basel). 2022;14(19).
4. Lam SW, Belova T, Franceschini N, van den Akker B, van IDG, Mikkers HM, et al. Truncated FOS impairs osteogenic differentiation and induces prostaglandin and NFkappaB signalling in an in vitro cell-of-origin model for osteoid osteoma and osteoblastoma. J Pathol. 2026;268(3):263–75.
