# Supplementary material for: Targeting vulnerabilities in IDH mutant tumours: The model matters
Source: Neoplasia. 2026 Jul 27;80:101338. doi: 10.1016/j.neo.2026.101338 (PMC13446203; doi:10.1016/j.neo.2026.101338)
Supplement: Supplementary file 3 [file mmc3.pdf]

Supplementary Tables

Supplementary Table S1. crRNAs and homology arms used in this study.

Target	Sequence
crRNA_IDH1 WT	TGGGTAAAACCTATCATCATAGG
crRNA_IDH1 R132G	GTAAAACCTATCATCATAGGTGG
Homology arm_IDH1 WT to R132C	AAAATATCCCCCGGCTTGTGAGTGGATGGGTAAAACCAATTATAATCGGGTGTCATGCTTATGGGGATCAAGTAAGTCATGTTGGCAATAATGTGATTTTG
Homology arm_IDH1 R132G to R132R	AAAATATCCCCCGGCTTGTGAGTGGATGGGTAAAACCAATTATAATCGGGCGTCATGCTTATGGGGATCAAGTAAGTCATGTTGGCAATAATGTGATTTTG

Underlining = PAM sequence; dotted underlining = inserted PsiI restriction site; green highlighting = silent mutations; red highlighting = R132 variants

Supplementary Table S2. Antibodies used in this study.

Target	Antibody	Dilution	Catalog number	Supplier
Vector-based IDH1^{R132C} and EGFP	2A peptide (T2A/P2A), clone 3H4	1:1,000	MABS2005	Merck, Darmstadt, Germany
Total IDH1 (endogenous and vector-based IDH1^{WT} and/or IDH1^{MUT})	IDH1	1:1,000	#3997	Cell Signaling Technology (CST), Leiden, the Netherlands
ATM	ATM, clone D2E2	1:500	#2873	CST
PARP	PARP, clone 46D11	1:1,000	#9532	CST
Mcl-1	Mcl-1	1:1,000	#4572	CST
Bcl-xL	Bcl-xL, clone 54H6	1:1,000	#2764	CST
Bcl-2	Bcl-2, clone D55G8	1:1,000	#4223	CST
Bcl-w	Bcl-w, clone 31H4	1:1,000	#2724	CST
Bak	Bak, clone D4E4	1:1,000	#12105	CST
Bax	Bax, clone D2E11	1:1,000	#5023	CST
Bim	Bim, clone C34C5	1:1,000	#2933	CST
Bid	Bid	1:1,000	#2002	CST
Bok	Bok, clone D7V2N	1:1,000	#86875	CST
GAPDH	GAPDH, clone D16H11	1:3,000	#5174	CST
α-tubulin	α-tubulin, clone DM1A	1:30,000	#3873	CST

Supplementary Table S3. IDH1 mutations in CH2879 and JJ012 cell lines around CRISPR-Cas9 target site.

Cell line	Genomic location	DNA	Protein	VAF (%)	Total reads	Consequence	Note
CH2879 Cr45-C5	chr2:208248389	G/A	R132C	24	108	Missense SNV	All SNVs are observed in the same reads, meaning SNVs co-occur
	chr2:208248390	A/C	G131G	24	108	Synonymous SNV	
	chr2:208248393	T/G	I130I	24	108	Synonymous SNV	
	chr2:208248396	G/T	I129I	24	109	Synonymous SNV	
	chr2:208248399	G/A	I128I	23	112	Synonymous SNV	
	chr2:208248402	A/T	P127P	23	114	Synonymous SNV	
	chr2:208248394-208248399	TATGATG/T	III/I (128-130)	26	108	Inframe deletion	
CH2879 Cr47-G4	chr2:208248389	G/A	R132C	22	104	Missense SNV	All SNVs are observed in the same reads, meaning SNVs co-occur
	chr2:208248390	A/C	G131G	22	105	Synonymous SNV	
	chr2:208248393	T/G	I130I	22	104	Synonymous SNV	
	chr2:208248396	G/T	I129I	22	104	Synonymous SNV	
	chr2:208248399	G/A	I128I	22	104	Synonymous SNV	
	chr2:208248402	A/T	P127P	24	107	Synonymous SNV	
	chr2:208248396-208248409	TGATGATAGGTTTTA/T	VKPII/X (125-130)	36	103	Frameshift deletion	
JJ012 M1-D3	chr2:208248389	G/C	R132G	32	81	Missense SNV	
JJ012 M1-D9	chr2:208248389	G/C	R132G	39	88	Missense SNV	
JJ012 M5-G11	chr2:208248389	G/C	R132G	45	101	Missense SNV	
JJ012 M7-E6	chr2:208248389	G/C	R132G	17	77	Missense SNV	

JJ012 Parental	chr2:208248389	G/C	R132G	31	87	Missense SNV	
JJ012 Cr2-C6	chr2:208248390	A/C	G131G	45	101	Synonymous SNV	All SNVs are observed in the same reads, meaning SNVs co-occur
	chr2:208248393	T/G	I130I	45	100	Synonymous SNV	
	chr2:208248396	G/T	I129I	44	103	Synonymous SNV	
	chr2:208248399	G/A	I128I	44	105	Synonymous SNV	
	chr2:208248402	A/T	P127P	44	108	Synonymous SNV	

Supplementary Table S4. Top 10 affected pathways in CRISPR-edited CH2879 and JJ012 IDH^{WT} and $IDH1^{MUT}$ cell lines.

Cell lines	Pathway	Rich Factor	Fold Enrichment	Adjusted p-value
CH2879 IDH^{WT}<br><br>M1-E3, M1-G5, M1-F10, and M1-B11	Focal Adhesion	0.228	1.619	0.101
	EGFR Tyrosine Kinase Inhibitor Resistance	0.274	1.947	0.108
	GABA receptor Signaling	0.333	2.370	0.212
	Pathways Regulating Hippo Signaling	0.242	1.724	0.212
	Robo4 and VEGF Signaling Pathways Crosstalk	0.667	4.741	0.212
	Regulation of Microtubule Cytoskeleton	0.286	2.032	0.231
	Phosphoinositides metabolism	0.265	1.887	0.373
	TGF-beta Signaling Pathway	0.211	1.497	0.373
	GPR40 Pathway	0.375	2.667	0.373
	Regulation of sister chromatid separation at the metaphase-anaphase transition	0.375	2.667	0.373
CH2879 $IDH1^{MUT}$<br><br>Cr45-C5 and Cr47-G4	Phosphoinositides metabolism	0.306	2.690	0.051
	DNA IR-double strand breaks and cellular response via ATM	0.236	2.077	0.504
	Resistin as a regulator of inflammation	0.273	2.397	0.504
	DNA IR-damage and cellular response via ATR	0.207	1.822	0.504
	SRF and miRs in Smooth Muscle Differentiation and Proliferation	0.385	3.380	0.504
	Regulation of toll-like receptor signaling pathway	0.172	1.515	0.749
	GPR40 Pathway	0.313	2.746	0.749
	ID signaling pathway	0.313	2.746	0.749
	ATM Signaling Pathway	0.225	1.977	0.749
	DNA damage response (only ATM dependent)	0.174	1.528	0.749

JJ012 IDH^{WT}<br><br>Cr2-C6, Cr31-E3, and Cr31-G11	IL-3 signaling pathway	0.041	10.900	0.268
	VEGFA-VEGFR2 Signaling Pathway	0.011	3.042	0.268
	EGF/EGFR signaling pathway	0.018	4.885	0.268
	AMP-activated protein kinase (AMPK) signaling	0.029	7.741	0.268
	T-cell receptor (TCR) signaling pathway	0.022	5.869	0.310
	Cancer immunotherapy by CTLA4 blockade	0.067	17.804	0.310
	PI3K/AKT/mTOR - VitD3 signaling	0.045	12.139	0.310
	Angiopoietin Like Protein 8 Regulatory Pathway	0.015	4.046	0.310
	Selective expression of chemokine receptors during T-cell polarization	0.034	9.209	0.310
	Inflammatory Response Pathway	0.030	8.093	0.310
JJ012 IDH1^{MUT}<br><br>M1-D3, M1-D9, M5-G11, and M7-E6	Osteoblast Signaling	0.143	32.701	0.075
	Fatty acid beta-oxidation	0.059	13.465	0.164
	Overview of leukocyte-intrinsic Hippo pathway functions	0.056	12.717	0.164
	Osteopontin Signaling	0.077	17.608	0.439
	Osteoclast Signaling	0.063	14.307	0.439
	Complement system	0.020	4.624	0.439
	Neural Crest Differentiation	0.020	4.488	0.439
	Gastrin signaling pathway	0.018	4.016	0.439
	Fatty Acid Biosynthesis	0.045	10.405	0.439
	NRF2-ARE regulation	0.043	9.952	0.439

Supplementary Table S5. Primer pairs used in this study.

Target	Forward Primer	Reverse Primer
$IDH1^{WT}$ promoter with restriction sites	GCGTTAATTAAGTCAGGGCAAG	TTAACCGGTTTTCCTCTTCCC
$IDH1^{R132C}$ gene with restriction sites	GCGTCTAGAATGTCCAAAAAAATCAGTGG	TTAGGATCCAAGTTTGGCCTGAGCTAGTT
Sequencing of $IDH1^{WT}$ promoter insert	GCCAATTCTGCAGACAAATG	TGAACTTGTGGCCGTTTAC
Sequencing of $IDH1^{R132C}$ gene insert	GGTCCTGCTGGAGTTCGTGA	CATAGCGTAAAAGGAGCAACA
IDH1 CRISPR-Cas9 target site	CGTCAAATGTGCCACTATCACT	GTAAGGGGATCCTATTGTGCAG
